# mMaple: A Photoconvertible Fluorescent Protein for Use in Multiple Imaging Modalities

**DOI:** 10.1371/journal.pone.0051314

**Published:** 2012-12-11

**Authors:** Ann L. McEvoy, Hiofan Hoi, Mark Bates, Evgenia Platonova, Paula J. Cranfill, Michelle A. Baird, Michael W. Davidson, Helge Ewers, Jan Liphardt, Robert E. Campbell

**Affiliations:** 1 Biophysics Graduate Group, University of California, Berkeley, California, United States of America; 2 Department of Chemistry, University of Alberta, Edmonton, Alberta, Canada; 3 Department of NanoBiophotonics, Max Planck Institute for Biophysical Chemistry, Göttingen, Germany; 4 Institute of Biochemistry, Eidgenössische Technische Hochschule (ETH) Zurich, Zurich, Switzerland; 5 National High Magnetic Field Laboratory and Department of Biological Science, The Florida State University, Tallahassee, Florida, United States of America; 6 California Institute for Quantitative Biosciences (QB3), University of California, Berkeley, California, United States of America; 7 Bay Area Physical Sciences – Oncology Center, University of California, Berkeley, California, United States of America; 8 Physical Biosciences Division, Lawrence Berkeley National Laboratory, Berkeley, California, United States of America; 9 Department of Physics, University of California, Berkeley, California, United States of America; Cardiff University, United Kingdom

## Abstract

Recent advances in fluorescence microscopy have extended the spatial resolution to the nanometer scale. Here, we report an engineered photoconvertible fluorescent protein (pcFP) variant, designated as mMaple, that is suited for use in multiple conventional and super-resolution imaging modalities, specifically, widefield and confocal microscopy, structured illumination microscopy (SIM), and single-molecule localization microscopy. We demonstrate the versatility of mMaple by obtaining super-resolution images of protein organization in *Escherichia coli* and conventional fluorescence images of mammalian cells. Beneficial features of mMaple include high photostability of the green state when expressed in mammalian cells and high steady state intracellular protein concentration of functional protein when expressed in *E. coli*. mMaple thus enables both fast live-cell ensemble imaging and high precision single molecule localization for a single pcFP-containing construct.

## Introduction

A new generation of fluorescence microscopes is capable of imaging with nanometer-scale resolution. These “super-resolution” microscopes are now commercially available and poised to become standard fixtures in imaging facilities and laboratories worldwide [1], [2].

Single-molecule localization microscopy such as photoactivated localization microscopy (PALM) [3], stochastic optical reconstruction microscopy (STORM) [4], and fluorescence-PALM (f-PALM) [5] (collectively referred to hereafter as (f-)PALM/STORM) provide information on the positions of many individual fluorophores within the sample at high precision, producing an image with high resolution (∼25 nm laterally [3]–[5] and ∼10–50 nm axially [6]–[8]) and enabling sub-diffraction limit imaging of cellular ultrastructure and quantitative analysis of protein distributions [9]. The highest resolutions have been obtained in fixed samples [4]–[7], [10], [11] but technical improvements now allow living samples to be characterized with effective frame rates on the second(s) timescale [12], [13].

By comparison, SIM and stimulated emission depletion microscopy (STED) provide relatively high-speed image acquisition [14], [15] and also achieve sub-diffraction limit image resolution. The spatial resolution of linear SIM is twice that of a conventional microscope (i.e., ∼125 nm laterally and ∼250 nm axially for visible light) [16], [17], while STED obtains diffraction-unlimited resolution, achieving 40–70 nm resolution in three dimensions for biological samples labeled with fluorescent dyes and proteins [18]–[21]. Characterization of live-cell protein dynamics is more tractable with these techniques [16], [17], [19], [22], [23]; however, the highest resolutions are typically obtained with fixed samples [3]–[5], [7].

Since each super-resolution implementation has its own advantages and limitations, it would be advantageous to apply multiple imaging modalities to one sample. This would allow investigators to watch dynamic complexes assemble and move, while also allowing them to characterize the detailed organization of these complexes, without the need for distinct probes and labeling strategies. Different super-resolution imaging approaches would appear to require fluorescent probes with seemingly incompatible properties. (f-)PALM/STORM requires probes that can be switched with high contrast between two spectrally distinct states, including photoactivatable FPs [24], [25], photoswitchable FPs [26], photoconvertible (pc) FPs [27]–[31] and organic dyes [4], [10], [32]–[34]. By contrast, SIM and STED are compatible with many conventional fluorophores (e.g., enhanced green FP (EGFP)) and for these methods, high fluorophore brightness and photostability are necessary for achieving the highest resolutions.

Here we report a new green-to-red pcFP variant, known as mMaple, derived from the previously reported pcFP mClavGR2 [31]. Using the *E. coli* chemotaxis network as a model system [9], we demonstrate that mMaple protein fusions are functional and provide brighter green state fluorescence intensity in cells than either EGFP or the widely used pcFP, mEos2. Furthermore, we show that the combination of green-to-red photoconversion and high green state brightness allow mMaple to be used for both (f-)PALM/STORM and SIM. Single pcFP counting in individual bacterial cells by (f-)PALM/STORM reveals that a key contributor to the favorable properties of mMaple is a high intracellular concentration of properly folded (and therefore photoconvertible) mMaple fusion proteins. Due to its improved folding efficiency and photostability, mMaple has significant advantages over other proteins currently used for super-resolution microscopy.

## Results and Discussion

### Engineering and Characterization of mMaple, a New Green-to-red pcFP

**Figure 1:**
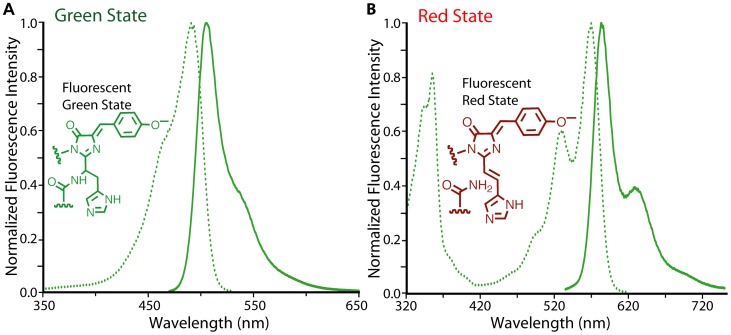
Spectral characterization of mMaple. (**A**) Excitation and emission spectra of the pre-photoconversion green state of mMaple. (**B**) Excitation and emission spectra of the post-photoconversion red state of mMaple.

Though mClavGR2 pcFP is monomeric at high concentrations [31], we were concerned that it might have a weak tendency to dimerize, since residues 220–224 (HSGLP) are identical to the ones that form part of the dimer interface in related *Anthozoa* FPs [35]. Thus, we replaced residues 220–224 with the corresponding residues (RNSTD) from the close homologue mTFP1 [36] (Fig. S1). Starting from this modified mClavGR2 variant, we undertook 4 rounds of protein optimization by creating successive libraries of ∼5 thousand genetic variants and screening these libraries for efficient photoconversion [31]. *E. coli* colonies with high brightness and an improved ratio of red fluorescence (after photoconversion) to green fluorescence (before photoconversion) were considered “winners” of a given round of screening. In the final round of optimization, the winners of all previous rounds were genetically shuffled [37]. Screening of this final library led to the discovery of mMaple, a variant that retains many of the key traits of mClavGR2 (**Fig. 1** **;** **Table 1**), yet provides an improved ratio of red-to-green photoconversion. mMaple is equivalent to mClavGR2 with the HSGLP to RNSTD replacement and A145V/G171S/G225S.

**Table 1:** Properties of mMaple and related variants.

Protein name	State	λ_ex_(nm)	λ_em_(nm)	ε^a^	Φ	Brightness^b^	p*K*_a_
mMaple	green	489	505	15 (26, 59)	0.74	11	8.2
mMaple	red	566	583	30	0.56	17	7.3
mClavGR2	green	488	504	19 (25, 60)	0.77	15	8.0
mClavGR2	red	566	583	32	0.54	17	7.3
mEos2	green	506	519	78 (32, 95)	0.43	34	5.6^c^
mEos2	red	573	584	39	0.35	14	6.4^c^

a Extinction coefficent (mM^−1^ cm^−1^) at peak absorbance wavelength in PBS (pH 7.4). Value in parentheses was determined at pH 4 and pH 10, respectively.

b Product of ε and Φ in mM^−1^ cm^−1^. For comparison, the brightness of EGFP and mCherry are 34 mM^−1^ cm^−1^ and 16 mM^−1^ cm^−1^, respectively [53].

c Data from McKinney *et al.* [30].

**Figure 2:**
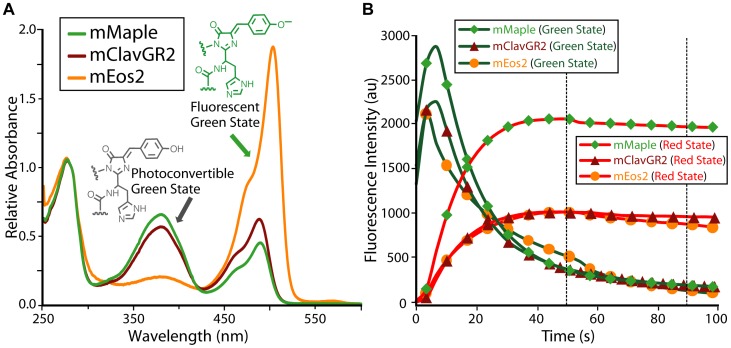
Characterization of mClavGR2 and its improved variants. (**A**) Absorbance spectra of mMaple (green line), mClavGR2 (red line) and mEos2 (orange line). Absorbance spectra are normalized to absorbance at 280 nm. (**B**) Photoconversion from the green state (green lines) to the red state (red lines) of mMaple (diamonds), mClavGR2 (triangles), and mEos2 (circles) for pcFP-H2B fusions expressed in HeLa S3 cells. The red-to-green contrast has been calculated at 47 s and 91 s (indicated with vertical dotted lines). At 47 s the contrast is 5.3 for mMaple, 2.1 for mClavGR2, and 1.8 for mEos2. At 91 s the contrast is 10.8 for mMaple, 4.7 for mClavGR2, and 6.9 for mEos2.

*In vitro* characterization revealed that the primary difference between mMaple and the earlier mClavGR2 variant is a shift in the ground state equilibrium of the green state chromophore from the phenolate form (absorbance λ_max_ = 489 nm) towards the phenol form (absorbance λ_max_ = 380 nm) (**Fig. 2A**). This shift is attributed to an increase in the apparent p*K*_a_ of the green state chromophore from 8.0 to 8.2 (**Table 1** **; Fig. S2**). The increased ratio of phenol to phenolate form in the green state explains the improved photoconversion contrast of mMaple (**Fig. 2**), since it is the phenol form that undergoes the green-to-red photoconversion and it is the phenolate form that is green fluorescent. The post-conversion red state retains the same p*K*_a_ as mClavGR2 (7.3), so the population of the red fluorescent phenolate form remains unchanged (**Table 1**). We speculate that the A145V mutation is primarily responsible for the shift of the green state p*K*_a_, since position 145 is located immediately adjacent to the tyrosine-derived phenolate moiety of the chromophore. Although it does not directly interact with the chromophore, the bulkier side chain of valine may stabilize the protonated state by decreasing the solvent accessibility of the chromophore. Notably, position 145 is occupied by proline in all other pcFPs except Kaede [27], which has alanine at this position. The effect of the additional mutations (G171S, G225S) is unclear, as they are relatively remote from the chromophore.

### *In vivo* Performance of mMaple in (f-)PALM/STORM Imaging

The basis of (f-)PALM/STORM imaging is the conversion of a fluorophore between two spectrally distinct states. We hoped that mMaple’s more highly populated photoconvertible green state would increase the number of observed proteins in (f-)PALM/STORM images, when compared to other pcFPs. We used the well-characterized *E. coli* chemotaxis network as a model system, due to its controllable expression levels and a sensitive functionality assay [9]. We prepared plasmids encoding fusions to the *E. coli* chemotaxis protein CheW under control of an L-arabinose inducible promoter. When expressed in a *cheW* knockout strain, each pcFP-CheW fusion recovers the strain’s chemotaxis ability to approximately 65% that of wildtype swarming (**Fig. S3A–B**).

**Figure 3:**
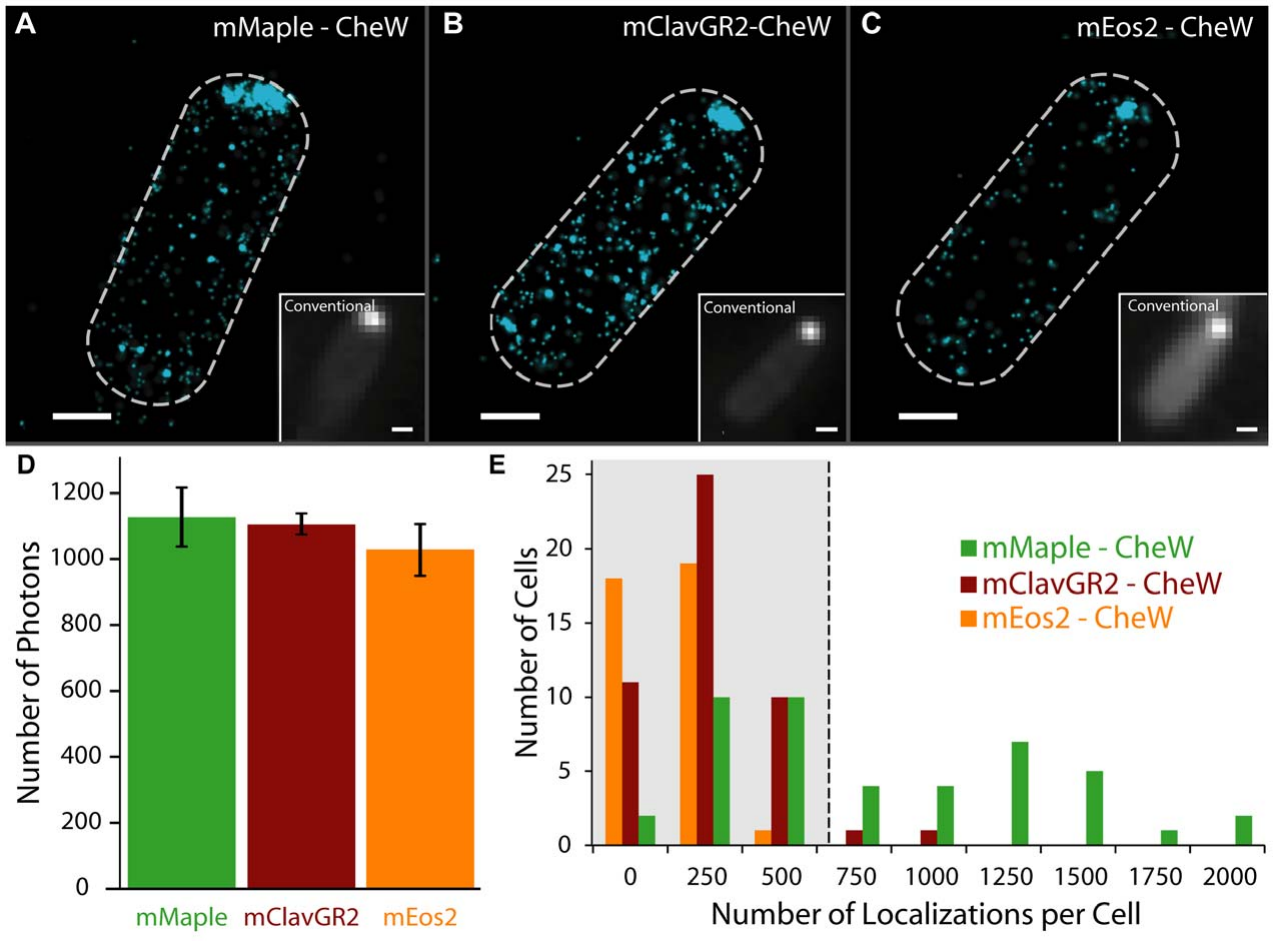
(f-)PALM/STORM comparison of mMaple, mClavGR2 and mEos2. (**A–C**) Images of Δ*cheW E.coli* expressing CheW fusion proteins at L-arabinose concentrations optimal for swarming. Images contain (**A**) 1086 mMaple-CheW localizations (**B**) 694 mClavGR2-CheW localizations and (**C**) 229 mEos2-CheW localizations. (**D**) The mean number of photons emitted by each construct per photoconversion event (error is standard error, *N* = 3 independent measurements from distributions consisting of 4,000–32,000 localizations). Scale bars are 500 nm. (**E**) Distribution of the number of localizations observed for Δ*cheW E. coli* cells containing CheW fusions to mMaple, mClavGR2, and mEos2. Greater than 96% of cells expressing either mEos2- or mClavGR2-CheW fusions have less than 500 localizations (boxed region), whereas greater than 50% of cells expressing mMaple-CheW fusions have more than 500 localizations.

**Figure 4:**
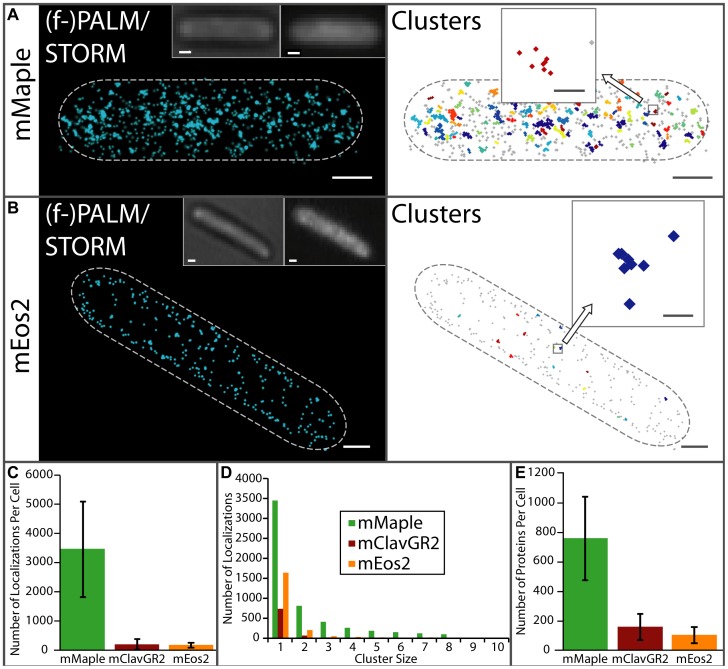
(f-)PALM/STORM characterization of the number of observed localizations and proteins per cell. (**A–B**) (f-)PALM/STORM images of fixed *E. coli* expressing cytoplasmic (**A**) mMaple (*N* = 1696 localizations), or (**B**) mEos2 (*N* = 472 localizations). Localizations are represented as normalized 2D Gaussian peaks with widths given by their theoretical localization precisions (left panels) and plotted as small markers grouped into clusters with adjacent spacing of 30 nm or less (right panels). Individual protein localizations are shown in grey whereas closely spaced localizations (<30 nm) are grouped into clusters of the same color (right panels). The bright field and conventional fluorescence images are shown for comparison (left panels, left and right inset respectively). Scale bars are 500 nm. (**C**) Average number of localizations per cell for each cytoplasmically expressed pcFP. (**D**) The distribution of cluster sizes (<30 nm interlocalization spacing) for cytoplasmically expressed pcFPs. (**E**) Average number of cytoplasmically expressed proteins per cell. Rather than counting each localization as a single molecule, we count each cluster of localizations (localizations spaced <30 nm) as a single protein. The dotted lines in (A–B) denote the *E. coli* cell boundary. Scale bars are 500 nm and 50 nm (zooms). Zooms in (A–B) show possible reversible photoswitching events of single proteins. Error is the standard deviation (N = 20 cells (mMaple), N = 17 cells (mClavGR2), N = 16 cells (mEos2)). The large error bars are primarily due to variation in protein expression between cells.

To quantify the number of proteins observed for each construct, we imaged fixed Δ*cheW E. coli* expressing CheW fusions to mMaple, mClavGR2, or mEos2 at optimal induction levels using a custom built (f-)PALM/STORM microscope [38]. In all 3 cases, (f-)PALM/STORM images revealed that the subcellular CheW distribution could be constructed from localizing hundreds of single proteins per cell (**Fig. 3A–C**). After photoconversion, the mean number of photons detected in the red state for each construct were similar, allowing for similar localization precision for all constructs [39] (**Fig. 3D** **; Fig. S4**). The largest difference among the pcFPs was that mMaple constructs reliably yielded more protein localizations than mEos2 or mClavGR2 per cell (3.4× and 2.3×, respectively) under identical growth and imaging conditions. At native expression levels, each cell should contain approximately 6000 CheW proteins [40]. However, since the depth of field of the objective restricts the observable region to the lower ∼40% of the cell, approximately 2400 CheW proteins should be imaged. On average, per-cell, we observed 927±547 mMaple-CheW localizations (*N* = 45 cells), 396±181 mClavGR2-CheW localizations (*N* = 48 cells), and 269±113 mEos2-CheW localizations (*N* = 38 cells) (**Fig. 3E**). The number of observable mMaple-CheW localizations is closer to the expected native levels of CheW expression than either mClavGR2 or mEos2. To ensure that the higher number of localizations is not fusion specific, we imaged each pcFP inside fixed wildtype *E. coli* with no binding partner (**Fig. 4A–B** **; left panels**). In this case, we obtain approximately 10× the number of localizations per cell for mMaple expressing cells (3497±1641 localizations) relative to mEos2 expressing cells (209±86 localizations).

It is important to note that measuring absolute numbers of proteins with (f-)PALM/STORM imaging is highly challenging. Factors such as non-fluorescent misfolded proteins, as well as on-off fluorescence switching events, in which fewer than 150 photons are emitted (below the reconstruction algorithm’s detection threshold), result in a fraction of fluorophores which are not counted, thus affecting the total number of localizations observed. Additionally, due to the relatively high p*K*_a_ of mMaple, at any one time a fraction of the photoconverted proteins will be in the protonated state and thus not observable in the red fluorescent channel. We expect that the interconversion of the protonated and non-protonated states is fast relative to our exposure times. This will cause each single fluorescent protein to spend part of the time in the red fluorescent state during each exposure. However, it is possible that these factors may lead to underestimations of the true numbers of fluorescent proteins in a sample.

### *In vivo* Performance of mMaple in SIM Imaging

**Figure 5:**
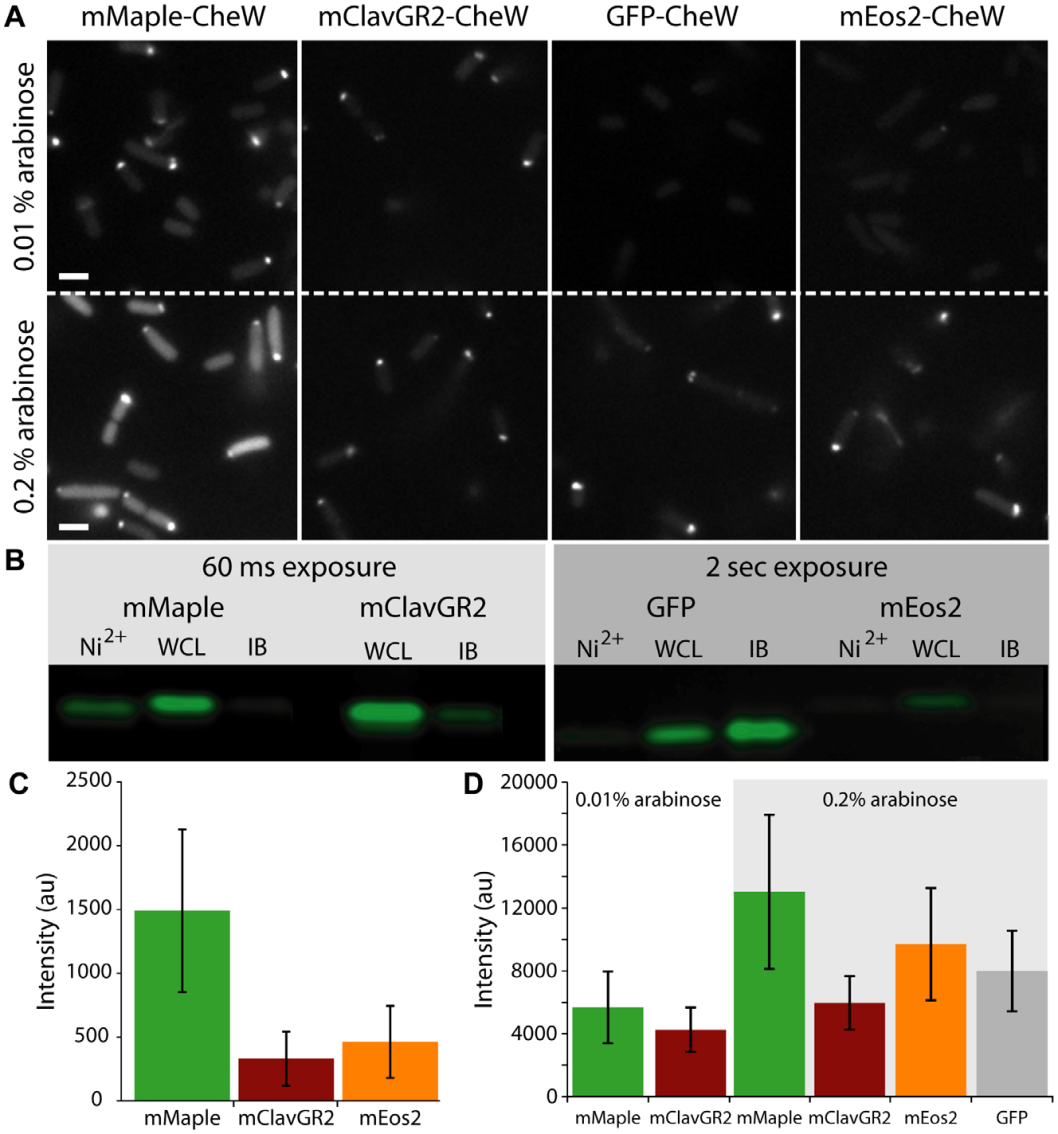
Green state fluorescence comparison between pcFP-CheW fusions. (**A**) *E. coli* was transformed with plasmids encoding CheW fusion proteins and cultures were induced with 0.01% L-arabinose (top row) and 0.2% L-arabinose (bottom row) for 3 h at 30°C and then imaged using 488 nm excitation. Note the brightness of the cells expressing mMaple fusions and the large percentage of cells with the correct polar localization pattern. (**B**) Fluorescence image of the bands corresponding to pcFP-CheW fusions extracted from *E. coli* and analyzed by SDS-PAGE gel. Proteins were either purified from the soluble fraction by Ni^2+^-NTA, solubilized from inclusion bodies with urea (IB), or loaded onto the gel as a whole cell lysate (WCL). Exposure times were increased from 60 ms to 2 sec in order to visualize the fluorescence of GFP-CheW and mEos2-CheW (right). Relative fluorescence intensities after correction for exposure times are: 1.00, 2.56, 0 (mMaple-CheW), 4.79, 0.43 (mClavGR2-CheW), 0, 0.08, 0.14 (EGFP-CheW), 0, 0.03 and 0 (mEos2-CheW). The Coomassie stained version of this gel is shown in **Fig. S2C**. (**C**) Cells expressing only cytoplasmic versions of each pcFP were grown in LB and induced with 0.002% L-arabinose for 3 hours. The mean fluorescence intensity for cells expressing mMaple was more than three times that of mEos2 (1489±636 for mMaple in comparison to 463±281 for mEos2) (*N* = 58 cells for mMaple, *N* = 57 cells for mClavGR2 and mEos2. (**D**) Mean fluorescence intensity for cells expressing each pcFP-CheW fusion. Consistent with the images in (A) and (B), at both induction levels, the mean fluorescence intensity level of mMaple-CheW is higher than all other CheW fusions (for 0.01% arabinose conditions, the number of cells analyzed was 69 for mMaple-CheW and 46 for mClavGR2-CheW respectively; for 0.2% conditions, *N* = 91, 62, 40, 71 cells for mMaple, mClavGR2, mEos2 and GFP fusions respectively).

In addition to proving that mMaple was suitable for (f-)PALM/STORM, our initial experiments also revealed that the mMaple-CheW construct had an exceptional green state brightness relative to both mEos2 and EGFP *in vivo*. Additionally, mMaple-CheW maintained the correct localization pattern at high concentrations (**Fig. 5A**), suggesting that protein mislocalization and inclusion body formation due to aggregation or misfolding was not limiting the potential utility of this protein. To further investigate the possibility of misfolding or inclusion body formation of pcFP-CheW fusions, we performed polyacrylamide gel electrophoresis (PAGE) under non-denaturing conditions. We found that although the total amount of soluble protein was similar for all pcFP-CheW fusions investigated (**Fig. S3C**), a larger fraction of the mMaple protein had reached the fully mature green state than either EGFP or mEos2 (**Fig. 5B**).

**Figure 6:**
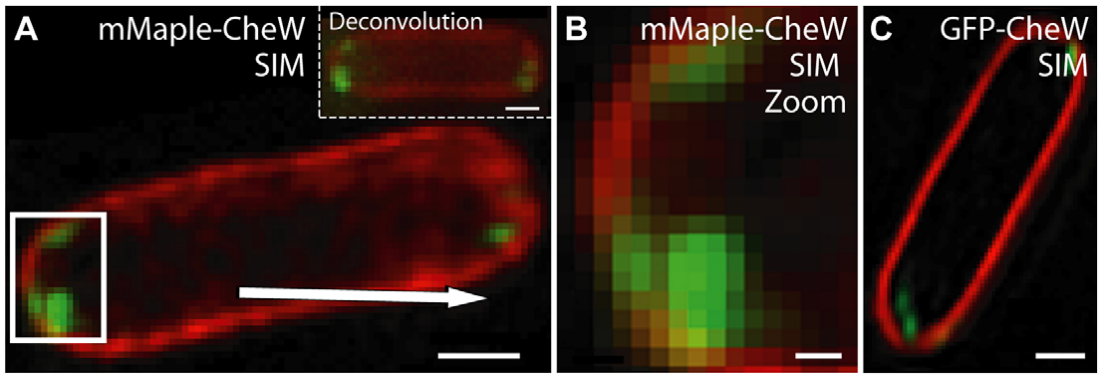
mMaple has improved *in vivo* brightness and enables 3D-SIM reconstructions. (**A**) Δ*cheW E.coli* expressing mMaple-CheW and a zoom (**B**) of the polar region of the cell denoted by the boxed region in (A). (**C**) One 125 nm slice of the 3D-SIM reconstruction of a Δ*cheW E. coli* expressing GFP-CheW. Red represents fluorescence of the membrane-specific dye FM4-64 and green represents FP fluorescence. Scale bars are 500 nm (A, C) and 100 nm (B).

The high *in vivo* green-state brightness of mMaple-CheW (**Fig. 5C–D**) motivated us to attempt 3-dimensional SIM reconstructions of live *E. coli* expressing either mMaple-CheW or EGFP-CheW (**Fig. 6** **; Movies S1, S2**). For the first time in our laboratories, we were able to acquire feature-rich SIM images with a pcFP. To produce SIM images of similar quality with EGFP, we needed to express the EGFP-CheW construct at 100× higher induction levels, which is consistent with the optimal induction levels for swarming for both constructs (**Fig. S3A-B**). This difference in necessary induction levels strongly suggests that mMaple is less disruptive than EGFP with respect to the folding and function of a genetically fused CheW partner. Attempts to acquire analogous data sets with mEos2-CheW were unsuccessful due to rapid photobleaching of the green state (**Figs. S5, S6; Table S1**), even though the initial green fluorescence intensities were similar to those of mMaple and EGFP (**Fig. 5D**).

### Origins of mMaple’s Superior Performance in both (f-) PALM/STORM and SIM

The experiments discussed above demonstrate that mMaple is a useful “multimodal” FP, but questions remained as to why mMaple protein fusions yielded higher numbers of localizations than other FPs in (f-)PALM/STORM images.

One possible explanation would be that mMaple has a higher propensity for reversible photoswitching, which would increase the number of observed localizations per cell. For instance, the red fluorescent state of mEos2 can undergo multiple cycles of reversible photoswitching into a long-lived dark state [41]. This effect is important to consider for accurate protein counting measurements using (f-)PALM/STORM images [9], [41], [42]. Indeed, we found that mMaple and mClavGR2 also exhibit reversible photoswitching of the red state (**Fig. S7**). To quantify the extent of reversible photoswitching, we used a previously described clustering algorithm [9] to group closely spaced protein localizations (<30 nm interlocalization spacing, **Fig. 4A–B**; right panels), and classified these groups as resulting from reversible switching of a single protein. Analysis of the (f-)PALM/STORM images of *E. coli* with cytoplasmically expressed pcFPs revealed that 56% of mMaple, and over 80% of both mClavGR2 and mEos2 localizations did not have a second localization within 30 nm (**Fig. 4D** **; Fig. S8**). To further quantify possible reversible switching events, we imaged single mMaple and mEos2 proteins immobilized on a glass coverslip using (f-)PALM/STORM (**Fig. S9A–B**). During image analysis, we once again grouped closely spaced localizations into clusters and obtained cluster size distributions for mMaple, mEos2, and the negative control for which no fluorescent proteins were present (**Fig. S9C**). The background subtracted cluster size distributions for each pcFP revealed that approximately 35% of mMaple localizations and 65% of mEos2 localizations were observed as single localizations (**Fig. S9D**). Therefore we conclude that, under our imaging conditions, the red fluorescent state of mMaple has a two-fold higher propensity to reversibly photoswitch than mEos2.

Does this two-fold increased reversible photoswitching completely account for the differences in the number of localizations observed in *E. coli* containing different cytoplasmically expressed pcFPs? We reassessed the (f-)PALM/STORM images of cytoplasmic pcFPs by counting both isolated localizations and clusters of localizations (<30 nm interlocalization spacing) as single proteins. Using this counting procedure we obtained an average number of proteins per cell for mMaple (765±283) that is approximately 7× higher than for mEos2 (109±55) (**Fig. 4E**), suggesting that the higher number of fluorophore localizations seen for mMaple constructs in *E. coli* is not simply due to reversible photoswitching.

We next investigated whether differences in photoconversion probability could explain the increased number of localizations observed for mMaple in (f-)PALM/STORM. The two factors that contribute to the photoconversion probability are the extinction coefficient at 405 nm and the quantum yield for photoconversion. The extinction coefficient of mMaple at 405 nm is 10,300 M^−1^ cm^−1^, whereas mEos2 is 4,400 M^−1^ cm^−1^. However, this 2.3× higher absorbance for mMaple is counterbalanced by a 5.5× lower quantum yield of photoconversion. Taking both factors into account, mEos2 has a 2.4× higher probability of photoconverting under identical illumination conditions. Accordingly, we conclude that the increased number of localizations obtained with mMaple is not due to differences in photoconversion probability.

A final possible contributor to the increased number of mMaple-CheW localizations observed in (f-)PALM/STORM images is a difference in pcFP folding/maturation efficiency in *E. coli*. We investigated this possibility *in vitro*, such that quantitative comparisons could be made. SDS-PAGE of the soluble and insoluble lysate fractions for *E. coli* expressing each pcFP revealed a substantial improvement in folding efficiency of mMaple (100% of mMaple was found in the soluble fraction compared to 29% for mEos2) (**Fig. S10**). An increased concentration of properly folded mMaple-CheW *in vivo* most likely accounts for both the increased number of localizations and the relatively low induction levels needed to provide strong green-state fluorescence signal (**Fig. 5A**). Does the increased folding efficiency of mMaple fully explain its performance in SIM imaging? Obtaining 3D-SIM reconstructions typically requires a large number of images of a sample, therefore it is important to use bright and photostable fluorophores to increase image quality. The high *in vivo* concentration of mMaple provides a partial explanation for the experimentally observed *in vivo* green-state brightness, but it does not explain the apparent lack of appreciable fading during prolonged SIM image acquisition. Quantifying resistance to photobleaching in living cells under widefield illumination, revealed that mMaple’s green state was 14-fold more photostable than mEos2’s green state, as judged by time to 50% loss of signal (65.1 sec for mMaple and 4.6 sec for mEos2) (**Fig. S5**). Therefore, we attribute the high quality of the *E. coli* SIM images to mMaple’s higher *in vivo* green state photostability. The advantages and limitations of mMaple relative to mEos2, for these specific applications and uses, are summarized in Table 2.

**Table 2:** Advantages and limitations of mMaple.

Imaging Method	mMaple Properties
Widefield epi-fluorescence, green state	∼14× higher photostability than mEos2
Widefield epi-fluorescence, red state	Similar photostability to mEos2
Confocal, green state	∼3.5× higher photostability to mEos2
Confocal, red state	∼2.4× higher photostability to mEos2
SIM, green state	Higher green-state photostability and higher fraction of properly folded proteins enhances image quality for *E. coli*
Single-molecule localization, (f-)PALM/STORM	Similar localization precision to mEos2. Twice as likely to reversibly photoswitch as mEos2.

### mMaple’s Performance in Mammalian Cells

**Figure 7:**
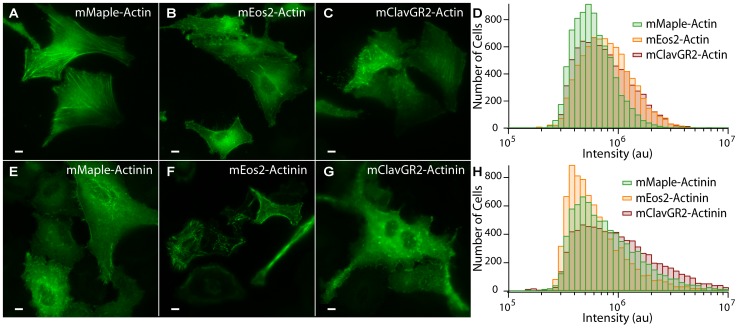
pcFP fusions expressed in mammalian cells. (**A**–**C**) Representative widefield fluorescence images of HeLa cells transfected with plasmids encoding either mMaple-actin (**A**), mEos2-actin (**B**), or mClavGR2-actin (**C**). (**D**) Flow cytometric analysis of the green fluorescence for HeLa cells transfected with the same plasmids used in A–C. A total of 7000 fluorescent cells were analyzed in each experiment. Relative median fluorescence intensity after excitation and emission correction is: 0.68 (mMaple), 0.77(mClavGR2) and 1.00 (mEos2). (**E**–**G**) Representative images of HeLa cells transfected with actinin fusions of mMaple (**E**), mEos2 (**F**), and mClavGR2 (**G**). (**H**) Flow cytometric analysis of the green fluorescence from HeLa cells transfected with the plasmids used in E–G. Relative corrected median fluorescence intensity is: 1.03 (mMaple), 1.25 (mClavGR2) and 1.00 (mEos2).

To determine mMaple’s performance in mammalian cells, we performed fluorescence imaging and flow cytometry analysis of HeLa cells transfected with plasmids encoding pcFP-actin and pcFP-actinin fusion proteins (**Fig. 7**). Visual examination of transfected cells revealed similar overall brightness and localization patterns for mMaple and mEos2. However, when the brightness of a large population of cells was assessed by flow cytometry, it was apparent that mMaple-actin or mMaple-actinin was not brighter than either of the corresponding mEos2 or mClavGR2 fusions when expressed in mammalian cells. For the actinin fusion, mClavGR2 had the greatest fraction of bright cells, followed by mMaple and mEos2. For the actin fusion, mClavGR2 and mEos2 had similar distributions that were shifted towards higher brightness relative to mMaple. Further examination of the actin constructs showed that though the overall brightness of the mMaple-actin constructs was not increased relative to the other pcFPs. Although these results were disappointing, we noted that the photostability of mMaple-actin is improved by almost three-fold (10.0 sec for mMaple in comparison to 3.6 sec as judged by confocal imaging), increasing the utility of the mMaple-actin construct in applications requiring green-state photostability (**Fig. S11**). Due to the large cell-to-cell heterogeneity in expression levels following transient transfection of mammalian cells, we did not attempt to compare numbers of localizations by PALM imaging. These results indicate that the exceptional folding efficiency of mMaple for prokaryotic imaging does not simply translate to mammalian cells. Perhaps, this limitation reflects the extensive directed evolution in bacterial cells used to generate mMaple. The brightness of mMaple and other pcFPs in mammalian cells is clearly fusion- and context specific and likely depends on a number of as-yet poorly understood factors. However, it is clear that the photostability of mMaple’s green state is much improved relative to mEos2 in both *E. coli* and mammalian cells.

### Conclusions

We have engineered a photoconvertible protein with a fortuitous combination of properties that enables this FP to be used in both (f-)PALM/STORM and SIM. The major advantages of mMaple over mEos2 are more efficient protein folding and a significantly increased photostability of the green state. We expect that this new variant will be of utility as it allows the dynamics of protein complexes to be characterized *in vivo* with increased spatial resolution and good temporal resolution (e.g., by SIM or conventional imaging), and also allows the precise localization of the same fusion proteins to be determined with (f-)PALM/STORM. It remains to be determined if these advantages will translate to other types of prokaryotic cells, and/or different mammalian cell fusions. We hope that the advent of mMaple will spur efforts to engineer a new generation of FPs that exploit the advantages of various imaging modalities.

## Materials and Methods

### General Methods and Materials

Primers were purchased from Integrated DNA Technologies (Coralville, IA) or Elim Biopharmaceuticals Inc. (Hayward, CA). The sequences of all primers used in this work are provided at the end of the Methods and Materials section. Plasmid and linear DNA purifications were performed using QIAprep spin miniprep or QIA gel extraction kits (Qiagen). All restriction enzymes were purchased from New England Biolabs. PCR amplifications were carried out using the PfuUltraII Fusion HS polymerase (Stragene) of Pfu polymerase (Fermentas). Sequencing reactions were performed using the BigDye Terminator v3.1 Cycle Sequencing Kit (Applied Biosystems) or with custom sequencing primers and analyzed at either the University of Alberta Molecular Biology Service Unit or UC Berkeley DNA Sequencing Facility. Mass spectrometry (MS) was performed by the MS Facility in the Department of Chemistry, University of Alberta.

### Engineering of Improved mClavGR Variants

A two-step PCR procedure was used to introduce the 220–224 HSGLP to RNSTD modification into mClavGR2. In the first step, the gene encoding mClavGR2 [31] was PCR amplified with the reverse primer1 and forward primer2. The resulting PCR product was used as the template for a second PCR amplification with forward primer2 and reverse primer3, which yielded the full length FP gene. As with all gene libraries for screening and single genes for large scale expression, purification, and *in vitro* characterization, the full length gene was digested by Xho1 and EcoR1 and ligated with T4 ligase (Invitrogen) into similarly digested pBAD/His B vector. To create a gene library with all possible residues at position 173, the template gene was subjected to two separate PCR amplifications: one with forward primer4 and reverse primer3 and one with forward primer2 and reverse primer5. The PCR products were combined and the full length gene assembled by overlap extension [43]. Creation of randomly mutated and gene shuffled libraries was carried out as previous described [31]. Following ligation, electrocompetent *E. coli* strain DH10B (Invitrogen) was transformed and plated on LB/agar plates supplemented with ampicillin (0.1 mg/ml) and L-arabinose (0.02%). For library screening, plates were incubated for 14 h at 37°C prior to inspection.

The screening setup and general protocol was been previously described [31]. Briefly, Petri dishes harboring colonies of *E. coli* transformed with a gene library were imaged to record their initial green fluorescence intensity. The plate was then subjected to illumination from a dense array of 405 nm LEDs. The red fluorescence intensity of the same dish was recorded after photoconversion. Digital fluorescence images were then processed to acquire both the green and red fluorescence intensity of each colony. In an effort to more effectively identify variants that exhibited rapid photoconversion, the illumination (i.e., photoconversion) time of the colony libraries was decreased from 20 min to 10 min. Colonies that exhibit both high brightness and an improved ratio of red fluorescence after photoconversion to green fluorescence before photoconversion are considered “winners” of a given round of screening and served as templates for the following round of library generation.

### *In vitro* Characterization of mMaple

Protein purification was carried out as previously reported [36]. The protein was exchanged into PBS (pH 7.4) buffer unless otherwise indicated. Molar extinction coefficients (ε) of the green states were measured by the alkali denaturation method and then used as reference to measure ε for the red states [44], [45]. To determine the ε of the red states, the pcFPs were photoconverted using 405 nm LED array until the red absorbance peak reached a maximum. Fluorescence quantum yields (Φ) were determined using fluorescein in 10 mM NaOH (Φ = 0.95) [46] and Rhodamine 6G in ethanol (Φ = 0.94) [47] as standards. The quantum yield of photoconversion (Φ_PC_) was determined using Φ_PC_ = [red]_1 min_/Int[I(x)×(1–10^−abs(x)^)dx ], where [red]_1 min_ is the concentration of red species after 1 min of photoconversion with the 405 nm LED, abs(x) and I(x) are the sample absorbance and illuminating light intensity at wavelength x, respectively, and the integral runs from 390 to 420 nm. The emission profile of the 405 nm LED used for photoconversion is defined as I(x) = I_0_ exp(-(x-405)^2^/36) based on the emission maximum and the full width at half maximum (FWHM) provided in the product catalogue. All absorption measurement was acquired on a DU-800 UV-visible spectrophotometer (Beckman). All fluorescence spectra were recorded on a QuantaMaster spectrofluorimeter (Photon Technology International) and have been corrected for the detector response. For determination of the pH dependence, purified protein in PBS was diluted 1∶50 into a series of pH-adjusted citrate saline (pH ≤ 8) or sodium phosphate (pH >8) buffers in a 96-well black clear bottom plate (Corning). Fluorescence was measured using a Safire2 plate reader (Tecan). The oligomeric structures of mClavGR2 and mMaple were determined by size-exclusion chromatography with a HiLoad 16/60 Superdex 75 pg gel-filtration column on a AKTA Prime Plus system (GE Health).

To measure the maturation profiles of mMaple, mClavGR2 and mEos2, *E. coli* transformed with pBAD/His B plasmids bearing the encoding cDNA was cultured overnight. The culture was diluted to an OD_600_ of 0.6, purged with argon for 20 min, sealed with a rubber septum, and incubated for another 1 h to allow thorough consumption of the residue oxygen. L-Arabinose (0.025%) was then added via a syringe with needle to induce expression of the FPs. After 4 h of incubation with shaking at 37°C, the cultures were transferred to an ice bath for 10 min and maintained at 4°C. Cells were centrifuged at 8000 rpm for 2 min, and cell pellets were lysed using vacuum-degassed B-PER II (Pierce) and incubated at room temperature for 10 min. The lysate was then centrifuged at 12,500 rpm for 5 min, and the supernatant was diluted 10× into PBS (pH 7.4). Fluorescence maturation was monitored at 37°C using a Safire2 plate reader (Tecan).

To evaluate the expression level of mMaple, mClavGR2 and mEos2, *E. coli* transformed with pBAD/His B plasmids bearing the encoding cDNA was cultured overnight. The culture was diluted to an OD_600_ of 0.6 in a total volume of 4 mL and L-arabinose (final concentration 0.02%) was added to induce the expression of the FPs. After 2 h growing at 37°C with shaking, the cells were collected, lysed with 50 µL of B-PER (Pierce), and centrifuged to separate the soluble proteins from insoluble material. An aliquot of the supernatant was taken for later SDS-PAGE analysis. The cell pellet was rinsed once with B-PER and once with 2 M urea. The pellet was then redissolved in 100 µL 8 M urea and centrifuged at 12000 rpm for 10 min. An aliquot of the supernatant was taken for later SDS-PAGE analysis. Both the supernatant from the lysate and the supernatant from the pellet extract were further purified by Ni^2+^-NTA-conjugated beads and all samples were analyzed by SDS-PAGE. The intensity of the bands was analyzed using ImageQuant RT ECL (General Electric).

Evaluation of the expression level of FP-CheW fusions was performed similarly except that (1) the *E. coli* strains used are RP437derivatives grown under the same culture conditions used for imaging (see below) and (2) 40 µg of each whole cell lysate was loaded onto the gel. Protein concentrations were determined using the BCA kit (Thermo Scientific). Fluorescent image of the gel were acquired using a custom built imaging system equipped with a 300 W Xeon lamp, a 450–490 nm filter for excitation and a 500–550 nm filter for emission. Fluorescent intensity was analyzed using Image Pro Plus (Media Cybernetics).

### Bacterial Strains and Plasmids for *E. coli* Imaging

Strains containing CheW fusions are derivatives of RP437, a chemotactic wild-type *E. coli* K-12 strain. CheW fusions were expressed in a strain lacking the genomic copy of CheW. All other strains are derivatives of MG1655, a wild-type *E. coli* K-12 strain. Strains derived from MG1655 contain the pJat plasmid [48], which contains the L-arabinose transporter gene (*araE*) under a constitutive promoter to increase the homogenous expression from the L-arabinose promoter. pJat is gentamicin resistant. All proteins were expressed from the inducible L-arabinose promoter on the low-copy plasmid pBAD (Invitrogen) containing a pBR322-derived origin, the ampicillin resistance gene (*bla*), and the a*raC* gene for positive regulation of the L-arabinose promoter. pALM1000 contains the EGFP gene only, pALM7000 contains the mEos2 gene only, pALM9000 contains the mClavGR2 gene only, and pALM10000 contains the mMaple gene only.

RP437 Δ*cheW* was made by P1 transduction from the Keio collection strains JW1876 (Δ*cheW::kan*). The deletion in this strain was constructed to minimize polar effects on downstream gene expression by retaining the native start codon and the last 18 C-terminal nucleotides [49].

### Construction of Plasmids for Expression of CheW Fusion Constructs

All fusions to CheW consist of the FP followed by the entire *cheW* gene (residues 1–167), and a terminal Glu-Phe encoding an EcoRI site. Plasmid pALM7000 was constructed by PCR amplification of the monomeric *Eos2* gene from the plasmid pRSETa_mEos2 (Addgene plasmid 20341) using primer6 and primer7, which contain NcoI and BamHI sites, respectively. The PCR product was inserted into plasmid pBAD (Invitrogen) according to the manufacturer’s instructions. The N-terminal plasmid leader sequence was removed by digestion with NcoI and religation. pALM9000 was constructed by PCR amplification of the monomeric *ClavGR2* gene from the plasmid pmClavGR2-C1 using primer8 and primer9 containing NcoI and PstI sites, respectively. The PCR product was subcloned into pALM7000 using the NcoI and PstI sites. pALM10000 was constructed by PCR amplification of the monomeric *Maple* (pALM10000) gene from the pBAD/HisB plasmid containing mMaple using primer10 and primer11. This PCR product was subcloned into pALM7000 using the NcoI and PstI sites. Plasmid pALM1000 was constructed by PCR amplification of EGFP from the pTrcHis2-EGFP plasmid using primer12 and primer13. This PCR product was subcloned into pALM7000 using the NcoI and PstI sites.

Fusions of pcFPs with CheW were constructed by PCR amplification of *cheW* from strain RP437 using primer14 and primer15, and cloned into the PstI and EcoRI sites of pALM1000, pALM7000, pALM9000, pALM10000, immediately after the FP gene to create pALM1001, pALM7001, pALM9001 and pALM10001 that contain *EGFP-cheW*, *mEos2-cheW*, *mClavGR2-cheW*, and *mMaple-cheW* gene fusions respectively.

### Construction of Plasmids for Expression in Mammalian Cells

To create the pcFP-actin constructs, we amplified the gene encoding pcFP with a 5′ primer with an NheI site and a 3′ primer with an XhoI site. The purified PCR products were then digested and ligated into pEGFP-actin (Clontech), whose FP-coding gene has been previously removed by the same restriction enzymes. pcFP-actinin constructs were generated in a similar way using a 5′ primer encoding an AgeI site, a 3′ primer encoding a BspEI, and the pactinin-EGFP vector (Clontech). Plasmid DNA for transfection was prepared using the GeneJET™ Plasmid Miniprep Kit (Fermentas) or using the Plasmid Maxi kit (QIAGEN, Valencia, CA).

### Preparation of Mammalian Cells for Imaging and Flow Cytometry

HeLa cells (CCL2 line; ATCC) were maintained in Dulbecco’s modified Eagle’s medium (DMEM) (Invitrogen) supplemented with 10% fetal bovine serum (FBS) (Sigma), 2 mM GlutaMax (Invitrogen) and penicillin-streptomycin. Transfection was carried using TurboFect™ (Fermentas) according to the manufacturer’s protocol. Imaging or cytometry experiment was conducted 36∼48 h after transfection. The growing medium was changed into HEPES (25 mM) buffered Hanks’ Balanced Salt Solution (HHBSS) for imaging. Imaging was carried on an inverted Nikon Eclipse Ti microscope equipped with a 150 W Lumen 200 metal halide lamp (Prior Scientific), a 60× oil immersion objective (Nikon), and a 16-bit 512SC QuantEM CCD (Photometrics). For cytometry experiment, cells were trypsinized, collected by centrifugation, resuspended in HHBSS and analyzed on a C6 Flow Cytometer (Accuri). About 7000 to 20000 events were recorded, depending on the transfection efficiency, but only the first 7000 events were plot in **Fig. 4**. At least two replicas were conducted for each constructs. Brightness corrected for excitation and emission was calculated via *F*_corr_ = *F*_app_/relative brightness* = F*_app_/(*EC*_488_×α ), where *F*_app_ is the average of the acquired median fluorescence intensity, *EC*_488_ is the FP’s extinction coefficient at 488 nm, and α is the fraction of the emission spectrum passing the given filter (515–545 nm).

### Preparation for in Depth Comparison of pcFP-actin Fusions in Mammalian Cells

Human carcinoma U2OS cells (HTB-96 line; ATCC) were plated on glass coverslips, and incubated overnight prior to transfection with either actin-mMaple, actin-mClavGR2 or actin-mEos2. Cells were transfected using Lipofectamine® 2000 Reagent. After 12 hours of expression live cells were imaged at a wavelength of 491 nm on the spinning-disk confocal microscope with CoolSNAP HQ2 CCD camera in the stream acquisition mode of MetaMorph software. To measure photobleaching, the normalized fluorescence intensity was logged over time with a temporal resolution of about 300 ms, and the half-life period t_½_ of the fluorophores was calculated using one phase decay equation. To test for statistical significant distributions of photobleaching times, we utilized the Mann-Whitney t-test. Differences between data points were considered significant at P≤0.0001.

### Determination of Photoconversion Rate and Photoconversion Contrast

HeLa S3 cells (CCL2 line; ATCC) were cultured in a 50∶50 mixture of Ham’s F-12 and Dulbecco’s modified Eagle’s medium (Invitrogen) supplemented with 12.5% fetal bovine serum (FBS). The cells were then seeded onto 35 mm Delta-T imaging dishes for live cell imaging under an atmosphere of 5% CO_2_. Cells were transfected in culture medium with Effectene (Qiagen) and 1 µg of purified plasmid DNA encoding the pcFP fused with human histone H2B. At 24 hours post-transfection, samples received fresh media and were imaged live. All photoconversion contrast measurements were performed on an Olympus FV1000 confocal microscope with an Olympus PLAPO 60× oil-immersion objective (NA = 1.4). FluoView software (Olympus) was used for microscope control and image acquisition, and Simple PCI software (Hamamatsu) was used for image analysis. The 488 nm argon and 543 nm helium-neon laser lines (Melles Griot) were used with a 405/488/543 dichroic mirror to excite the green and red forms of each protein. Emission was collected in two channels spanning 500–533 nm (488 nm laser) and 550–660 nm (543 nm laser). For photoconversion, a 405 nm diode laser line (Olympus Simultaneous scanner unit) was used with the same dichroic for stimulation. Cells were imaged at a scan speed of 4.0 µs/pixel and were stimulated with the Simultaneous Scanner at a speed of 2.0 µs/pixel. Each experiment was performed with a pinhole size of 600 µm.

A single relatively bright nucleus was selected for imaging and both the red and green fluorescence channels were imaged while the entire nucleus was stimulated using the 405 nm Simultaneous Scanner. The average intensity at each time point for a region-of-interest within the nucleus was determined in software for each of 10 independent experiments and the average value was plotted as a function of time. To assess the rate of photoconversion, the time at which red fluorescence reached half of its maximum value was determined. Photoconversion contrast was calculated as the ratio of red fluorescence intensity (arbitrary units) to green fluorescence intensity (arbitrary units) immediately after red fluorescence had reached a maximum and near the end of the experimental time course.

### Determination of Photobleaching Rates

Laser-scanning confocal and widefield microscopy photobleaching experiments utilized HeLa S3 cells (CCL2 line; ATCC) expressing fusions of pcFP fused with human histone H2B, as described above for the photoconversion experiments. Nuclei with similar size and fluorescence intensity were chosen for photobleaching experiments. For laser-scanning confocal photobleaching an Olympus FV1000 was used to first image the cells at a low magnification to ensure cell vitality. The microscope was set to a zoom of 8×, a photomultiplier voltage of 500 V, and an offset of 12%, with a scan time of 4 µs/pixel. Cells were photobleached utilizing an Olympus PLAPO 60× oil-immersion objective (NA = 1.4) using either a 488 nm (green state) or a 543 nm (red state) laser line that was maintained at an output power of 120 µW using a FieldMax II-TO (Coherent) power meter. The fluorescence signal was collected in two channels spanning 500–522 nm (488 nm laser) and 550–660 nm (543 nm laser). A 405 nm diode laser line was used to photoconvert the protein.

For widefield photobleaching experiments, transfected HeLa S3 cells (CCL2 line; ATCC) in a Bioptechs Delta-T imaging chamber were imaged on a Nikon TE2000 inverted microscope equipped with a 40× dry objective (Nikon Plan Fluorite NA = 0.85) and an X-Cite exacte light source (Lumen Dynamics). To ensure the same power levels were used for each filter set, a Newport 1918-C optical power meter was used at the objective to measure the illumination intensity. Power was moderated using neutral density filters contained in the lamp. Regions of the dish containing 10–20 nuclei were photobleached for 3000 frames at a 100 ms exposure time with no delay. Images were collected using a QImaging Retiga EXi camera (Photometrics). Photoconversion was conducted using an Omega QMax Blue filter set. Photobleaching was conducted using a Chroma FITC-HYQ cube (green species) and a Semrock TRITC-A-000 cube (red species) at a power of 11.4 mW/cm^2^. The raw data was collected using NIS-Elements software (Nikon) and analyzed with Simple PCI software (Hamamatsu).

### *E. coli* Cell Culture Conditions for Imaging

Strains derived from RP437 were grown overnight in T-broth (1% w/v Difco Bacto-Tryptone (Becton Dickinson and Company), and 0.5% w/v NaCl (Fisher-Scientific) (pH 7.0)) at 30°C with aeration. Day cultures were inoculated to an optical density at 600 nm (OD_600_) of approximately 0.01 into T-broth with appropriate antibiotics at 30°C with aeration until they reached an OD_600_ 0.1–0.3. Protein expression was induced by adding 0.01% or 0.02% L-arabinose for 3 hrs, as indicated. Media and temperature were chosen to obtain the highest expression levels of properly folded proteins [28], [30], [31], [50].

### Swarm Plate Assay

To assess the functionality of chemotaxis fusion proteins, 2 µl of stationary-phase cells were spotted on soft-agar swarm plates and incubated at 30°C for 5 h. Wild-type RP437 *E. coli* cells were compared with a CheW deletion strain and CheW deletion strains with fluorescently tagged CheW fusion proteins (cells used for imaging). All complemented strains contain plasmids derived from pBAD (pBAD TOPO-TA Invitrogen), which confers ampicillin resistance and is L-arabinose inducible. Swarm plates contain 0.3% agar (Becton-Dickinson) in T-broth supplemented with varying concentrations of L-arabinose. Cells were grown in tryptone broth with appropriate antibiotics at 30°C prior to spotting on swarm plates.

### Sample Preparation and Imaging Protocol for SIM

Cells were harvested by centrifugation at 2,000 *g* for 15 minutes. The outer membrane of the *E. coli* was fluorescently labeled with 10 ng/µL FM4-64 (Invitrogen) diluted in M9 (1.05% M9 salts (Amresco) supplemented with 2 mM MgSO_4_, 0.1 mM CaCl_2_ and 0.4% glycerol) for 5 minutes. Cells were washed twice with M9 media and resuspended in fresh M9 media. 20 uL of 1.5% low-melt agar (Apex) dissolved in M9 media was deposited on a 25 mm ×75 mm single shallow depression slide (Boreal Northwest) flanked by two pieces of double stick tape and allowed to air dry. 0.5 µL of cells labeled with FM4-64 were deposited on the top of the agar and sandwiched between a 22 mm^2^ #1.5 microscope coverslip (Fisherbrand).

SIM imaging was performed on the Deltavision|OMX V3.0 (Applied Precision Inc, Issaquah, WA) containing 405 nm, 488 nm, 514 nm, 593 nm and 642 nm laser lines [51]. The sample was imaged with a 100×1.40 NA oil objective with 1.514 or 1.516 index immersion oil. Eukaryotic cells were imaged with 488 nm excitation and *E. coli* cells were imaged with 488 nm and 594 nm excitation. The fluorescence emission was split by channel, filtered and imaged using a dedicated custom monochrome 20 MHz camera with Sony ICX285 ER progressive scan CCD using 5–60 ms exposures. Acquisition was controlled by the OMXN controller software (Applied Precision Inc, Issaquah, WA) while reconstructions were made with the OMX specific SoftWoRx v4.5.0 software package (Applied Precision Inc., Issaquah, WA). 3D reconstructions were obtained in 125 nm steps.

### Sample Preparation and Imaging Protocol for (f-)PALM/STORM

Cells were fixed harvested by centrifugation at 2,000 *g* for 15 minutes. Cells were resuspended in 2–4% paraformaldehyde (Electron Microscopy Sciences) with 0.1–0.2% gluteraldehyde (Electron Microscopy Sciences) in PBS (pH 7.4) for 10 minutes. Cells were washed twice with PBS and resuspended in fresh PBS. #1.5 Lab-TekII 8-well chambers (Nalge Nunc International) were covered with 200 µL 0.1% w/v poly-L-lysine for 15 min then rinsed with water. Cells were added and spun onto coverslips at 2,000 *g* for 10 min. The sample were then rinsed with PBS and left in fresh PBS for imaging.

(f-)PALM/STORM imaging was performed according to Greenfield *et al.* [9] on an Olympus IX71 inverted microscope equipped with a 100×, 1.40 NA objective (Olympus) [38]. 405 nm and 561 nm laser light was delivered to the microscope through free space. 488 nm light was delivered via a mercury lamp with appropriate excitation and emission filters. Single-molecule fluorescence signals generated during acquisition were separated from the activation and excitation light using appropriate filter sets [10], [52] within the microscope and passed to an electron-multiplying charge-coupled device (CCD) camera running at approximately 20 Hz (50 ms exposures). Movie acquisition times were dependent on the regions of highest labeled-protein density. Activation intensity was increased slowly such that a given diffraction-limited spot contained no activated proteins >90% of the time. This is necessary to ensure that only one protein is activated at a time in a single diffraction-limited spot. Image generation and data analysis were done using custom Matlab scripts (Mathworks), as described by Greenfield *et al.* [9], and custom IDL software [10].

The localization and image-rendering algorithms used in this work have been previously described [3], [4]. Briefly, images were filtered and proteins were identified as signals that contained counts larger than four standard deviations above background. Proteins that became dark, but reappeared within five frames, were counted as the same protein. Photon distributions were obtained from proteins emitting at least 300 photons. In the case of the *E. coli* (f-)PALM/STORM images, only proteins that emitted at least 150 photons were counted, for mammalian cell (f-)PALM/STORM images, only proteins that emitted 400 photons or more were included. Sample drift was corrected by previously described algorithms [3], [6], [10].

Single protein localizations were grouped into clusters using a tree-clustering algorithm [9]. Proteins spaced less than 30 nm apart from each other are considered to be part of the same cluster, where clusters contain 2 or more proteins. 30 nm interlocalization spacing was chosen because it is twice the mean localization precision for these pcFPs [9], [39].

### Sample Preparation and Imaging Protocol for (f-)PALM/STORM with Purified Protein

mMaple and mEos2 were purified as described above and then biotinylated using EZ-Link Sulfo-NHS-Biotin (Pierce) following the manufacturer’s instruction. This biotinylation kit would provide a spacer about 22.4 Å between the pcFP and the biotin module. The spectral profile of absorbance, excitation and emission of the pcFPs were measured and found to remain unchanged. The number of labeled biotins per pcFP molecule was determined to be a distribution that ranged from 7–13, based on MALDI-MS.

Single biotinylated pcFPs were immobilized on an glass coverslip by incubating the slide with 1.0 mg/mL biotinylated bovine serum albumin (b-BSA, Sigma) solution for 30 sec, followed by 0.25 mg/mL streptavidin (Invitrogen) and then biotinylated pcFPs at approximately 0.6 µM in PBS, which were sonicated prior to addition. To correct for drift, 200 nm yellow-green beads (Molecular Probes) diluted in PBS were added to the chamber and immobilized using a buffer containing 10 mM Tris, pH 7.5, 50 mM NaCl and 50 mM MgCl_2_. The slide was rinsed with PBS prior to the addition of each reagent.

Single pcFPs were imaged in PBS using continuous activation and excitation using 405 nm and 561 lasers for 10,000 frames at constant laser power. To determine the degree of false positive events observed during imaging, a sample chamber containing only PBS and fiduciary markers was imaged. (f-)PALM/STORM images were reconstructed and drift corrected as described above.

To determine the degree of reversible photoswitching, closely spaced molecules were grouped, resulting in a cluster size distribution for each pcFP as well as the negative control. The distribution obtained from the negative control was subtracted from the resulting pcFP distributions to remove the population of false positives resulting from events unrelated to pcFP fluorescence.

### List of primers

primer1∶5′-CAGCTCGTCCATGCCGTCGGTGGAGTTGCGGGCCACGGCGTG-3′.

primer2∶5′-GCAGGTGAGTAACTCGAGCATGGTGAGCAAGG-3′.

primer3∶5′-GCCGAATTCTTACTTGTACAGCTCGTCCAT-3′.

primer4∶5′-NNKCGCTGCGACTTCCGCACCTA-3′.

primer5∶5′-AAGTCGCAGCGMNNGTGGCCGCCGCC-3′.

primer6∶5′-GGATCCATGGGGGCGATTAAGCCAGAC-3′.

primer7∶5′-CAAGCTTCTTAGGATCCTCGTCTGGCATTGTCAGGC-3′.

primer8∶5′-CCGGTCGCCACCATGGTGAGCAAGG-3′.

primer9∶5′-GAGATCTGAGCTGCAGCTTGTACAGCTCG3′.

primer10∶5′-GCTCGACCATGGTGAGCAAGG-3′.

primer11∶5′-CCAAGCTTCGAACTGCAGCTTGTACAGCTC-3′.

primer12∶5′-GGAGGAATAAACCATGGTGAGCAAG-3′.

primer13∶5′-CGTAAGCTTCCTGCAGCTTGTACAGCTCG-3′.

primer14∶5′-AAAGGTCTGCAGATGACCGGTATGACGAATGTAAC-3′.

primer15∶5′-TCGGGAGAATTCCGCCACTTCTGACG-3′.

## Supporting Information

Figure S1 **Gel filtration chromatography of mMaple and mClavGR2.** Both mClavGR2 (injection concentration of 0.5 mM) and mMaple (injection concentration of 0.5 mM) purified from *E. coli* by Ni^2+^-NTA affinity chromatography show an additional peak at 63 min. This peak is diminished in size for mMaple relative to mClavGR2 (6.7% vs. 25.8% of monomer peak area). While this peak does elute at a time consistent with the dimer species, it was not observed following reinjection of the collected and concentrated monomeric peak of either mClavGR2 (injection concentration of 0.34 mM) or mMaple (injection concentration of 0.39 mM). The fact that the species eluting at 63 min was not observed in the reinjection suggests that it is not the typical non-covalent dimer species expected for weakly dimerizing fluorescent proteins. While the nature of this species remains unclear, it is apparent that the tendency of it to form is reduced in mMaple.
(TIF)

Figure S2 **pH titrations of pcFP variants.** For each variant the fluorescent intensity at pH values ranging from 5 to 10 was determined by diluting purified protein into concentrated buffer adjusted to the appropriate pH. For the green state (green line, diamond symbols), the λ_ex_ = 440 nm and the λ_em_ = 530 nm. For the red state (red line, triangle symbols), the λ_ex_ = 540 nm and the λ_em_ = 630 nm. (**A**) mMaple. (**B**) mClavGR2.
(TIF)

Figure S3 **Swarm plate assays to assess the function of CheW fusions.** Approximately 2 µl of Δ*cheW E. coli* transformed with a plasmid encoding a FP-CheW fusion were placed on T-broth soft agar swarm plates. The ability for the *E. coli* to undergo chemotaxis was assessed by measuring the diameter of the swarm ring 5 h after the bacteria were placed on the agar at 30°C. The Δ*cheW* strain has no apparent swarm ring and strain RP437 exhibits the wild-type swarm ring. Interestingly, fusions with all of the pcFPs used in this work are able to rescue the swarming phenotype more effectively than the analogous GFP fusion. (**A**) Swarming ability as a percentage of wild-type for bacteria expressing mMaple, mClavGR2, mEos2, or GFP fused to CheW. Excessively high concentrations of CheW can disrupt swarming ability, and thus the size of swarm rings will decrease at high inducer concentration [54]. Error bars are standard error, *N* = 3 measurements. (**B**) Image of a representative agar plate (0.01% L-arabinose concentration) showing swarm rings for each of the constructs mentioned above. (**C**) Coomassie stained SDS-PAGE gel of the soluble and insoluble fractions of *E. coli* expressing pcFP-CheWs (row denoted by red arrow) described in this work. First and last lanes are the protein ladder. Relative intensity of the bands is: 132, 120 and 27 for EGFP-CheW; 82, 52 and 0 for mEos2-CheW; 100, 164 and 0 for mMaple-CheW; 154 and 0 for mClavGR2; Samples were not denatured prior to loading.
(TIF)

Figure S4 **Number of photons emitted by pcFPs fused to CheW.** Representative distributions of the number of photons emitted in the red fluorescent state by CheW fusions to (**A**) mMaple (**B**) mClavGR2 and (C) mEos2. Only localizations emitting more than 300 photons were included.
(TIF)

Figure S5 **Widefield imaging photobleaching curves for pcFPs.** Photobleaching curves of the green state (left panels) and the red state (right panels) of pcFP-H2B fusions expressed in HeLa S3 cells with widefield illumination. Each curve represents the photobleaching behavior of an individual cell and the darker colored curve is the average. Average time when the fluorescence intensity of the green states (left panels) decreased to half of the initial intensity are 65.1 sec for mMaple (**A**) (45 cells), 69.7 sec for mClavGR2 (**B**) (42 cells), and 4.6 sec for mEos2 (**C**) (48 cells). The average time for the red state is 180.3 sec for mMaple (**A**) (36 cells), 241.2 sec for mClavGR2 (**B**) (35 cells), and 205.8 sec for mEos2 (**C**) (49 cells). Values have been tabulated in Table S1.
(TIF)

Figure S6 **Confocal imaging photobleaching curves for pcFPs.** Photobleaching curves of the green states (left panels) and the red states (right panels) of pcFP-H2B fusions expressed in HeLa S3 cells with confocal illumination. Each curve represents the photobleaching behavior of an individual cell and the dark colored curve is the average. Average time for the green state fluorescence intensity to decrease to half of the initial intensity are 9.4 sec for mMaple (**A**) (22 cells), 4.6 sec for mClavGR2 (**B**) (30 cells), and 2.7 sec for mEos2 (**C**) (28 cells). The average time for the red state fluorescence intensity to decrease by half is 133.2 sec for mMaple (**A**) (27 cells), 206.3 sec for mClavGR2 (**B**) (23 cells) and 55.1 sec for mEos2 (**C**) (25 cells). Values have been tabulated in Table S1.
(TIF)

Figure S7 **Reversible photoswitching of photoconverted (red) mMaple.** (**A**) Photoconverted mMaple can be further photoconverted to a “dark” non-fluorescent state by illumination with green light. (**B**) The dark state of the photoconverted mMaple reversibly photoswitches back to the red fluorescent state as seen by changes in the absorbance spectra in response to different light sources. The absorbance at 280 nm (purple line, corresponding to total protein concentration), the absorbance at 457 nm (blue line, corresponding to the dark post-conversion red state) and the absorbance at 566 nm (red line, corresponding to the red fluorescent state of the protein) are plotted. As the protein is exposed to 532 nm light, the protein is switched from the red state to the dark state (green regions), which can be re-excited by 460 nm light (blue region), 405 nm light (violet region) and white light (light grey region). No absorbance changes were observed if the protein was kept in the dark (dark grey region). Similar results were obtained for mClavGR2.
(TIF)

Figure S8 **Cluster analysis of pcFP localizations.** The percentage of localizations grouped into clusters (<30 nm interlocalization spacing) for cytoplasmically expressed pcFPs. Over 50% of mMaple and over 80% of mClavGR2 and mEos2 proteins do not have a second localization within 30 nm.
(TIF)

Figure S9 **(f-)PALM/STORM analysis of purified pcFPs.** (**A–B**) Composite images of purified (**A**) mMaple and (**B**) mEos2 proteins immobilized on a coverslip. Localizations are represented as normalized 2D Gaussian peaks (right half) and represented as single localizations and clustered markers (<30 nm interlocalization spacing) (left). Scale bars are 2 µm and 50 nm (zooms). (**C**) Cluster size distribution for purified mMaple and mEos2 as well as a no protein control. (**D**) False-positive corrected cluster size distributions for purified mMaple and mEos2 demonstrate that mMaple is approximately twice as likely to reactivate as mEos2. (**inset**) Percentage of false-positive corrected localizations found to be in clusters for purified pcFPs. 35% of mMaple and 65% of mEos2 do not have a second localization within 30 nm.
(TIF)

Figure S10 **Expression and maturation of new pcFPs expressed in** ***E*** **.** ***coli*** **.** (**A**) Maturation of mClavGR variants and mEos2 at 37°C. The maturation profiles of mMaple (green) and mClavGR2 (dark red) can be fit as monoexponential curves with time constants of 39 min and 29 min, respectively. Under the conditions of this experiment, mEos2 (orange) is approximately 50% as bright as mMaple and, in agreement with our previous results [31], appears to have fully matured prior to the initial measurement. Each curve represents the average of six independent measurements and error bars represent standard deviations. (**B**) SDS-PAGE of the soluble and insoluble fractions of *E. coli* expressing pcFPs described in this work. Lane 1 is the protein ladder. For each construct, one lane corresponds to the whole cell lysate (WCL) and the other lane corresponds to protein from inclusion bodies (IB). The relative intensity of FP bands in the WCL and IB fractions, respectively, are: 17 and 7 for mEos2; 100 and 0 for mClavGR2; 113 and 0 for mMaple. Overall, mMaple shows the highest expression and folding efficiency with 100% of the protein in the soluble fraction, while mEos2 has the lowest expression and folding efficiency with 29% of the total expressed protein located in the IB fraction. (**C**) The same samples as in (**B**), following purification by Ni^2+^/NTA affinity chromatography.
(TIF)

Figure S11 **Photostability and green-state brightness characterization of pcFP-actin fusions in mammalian cells.** Representative widefield fluorescence images of U2OS cells transfected with plasmids encoding either mMaple-actin (**A**), mClavGR2-actin (**B**), or mEos2-actin (**C**). The cells were stained with phallodin as a comparison (insets in A–C). (**D**) All pcFP-actin fusions have similar cellular intensities. However, mMaple is more photostable than the other two pcFP fusions (**E**). The data in (**E**) were found to be statistically significant using the Mann-Whitney *t*-test (p≤0.0001).
(TIF)

Table S1 **Characterization of photobleaching rates for pcFP-H2B fusions in live cells.**
(DOC)

Movie S1 **3D animation of a SIM reconstruction of many live** ***E. coli*** **cells expressing mMaple-CheW (top left channel) stained with the live-cell membrane stain FM4-64 (top right channel) (as shown in** **Fig. 3C–D** **).** The channels have been combined in the bottom right panel. Scale bar is 1 µm.
(MOV)

Movie S2 **3D animation of a SIM reconstruction of a live** ***E. coli*** **expressing GFP-CheW (as shown in** **Fig. 3E** **).** The cellular membrane was stained with FM4-64. Scale bar is 500 nm.
(MOV)
