# Supplementary figures and images for: mMaple: A Photoconvertible Fluorescent Protein for Use in Multiple Imaging Modalities
Source: PLoS One. 2012 Dec 11;7(12):e51314. doi: 10.1371/journal.pone.0051314 (PMC3519878; doi:10.1371/journal.pone.0051314)

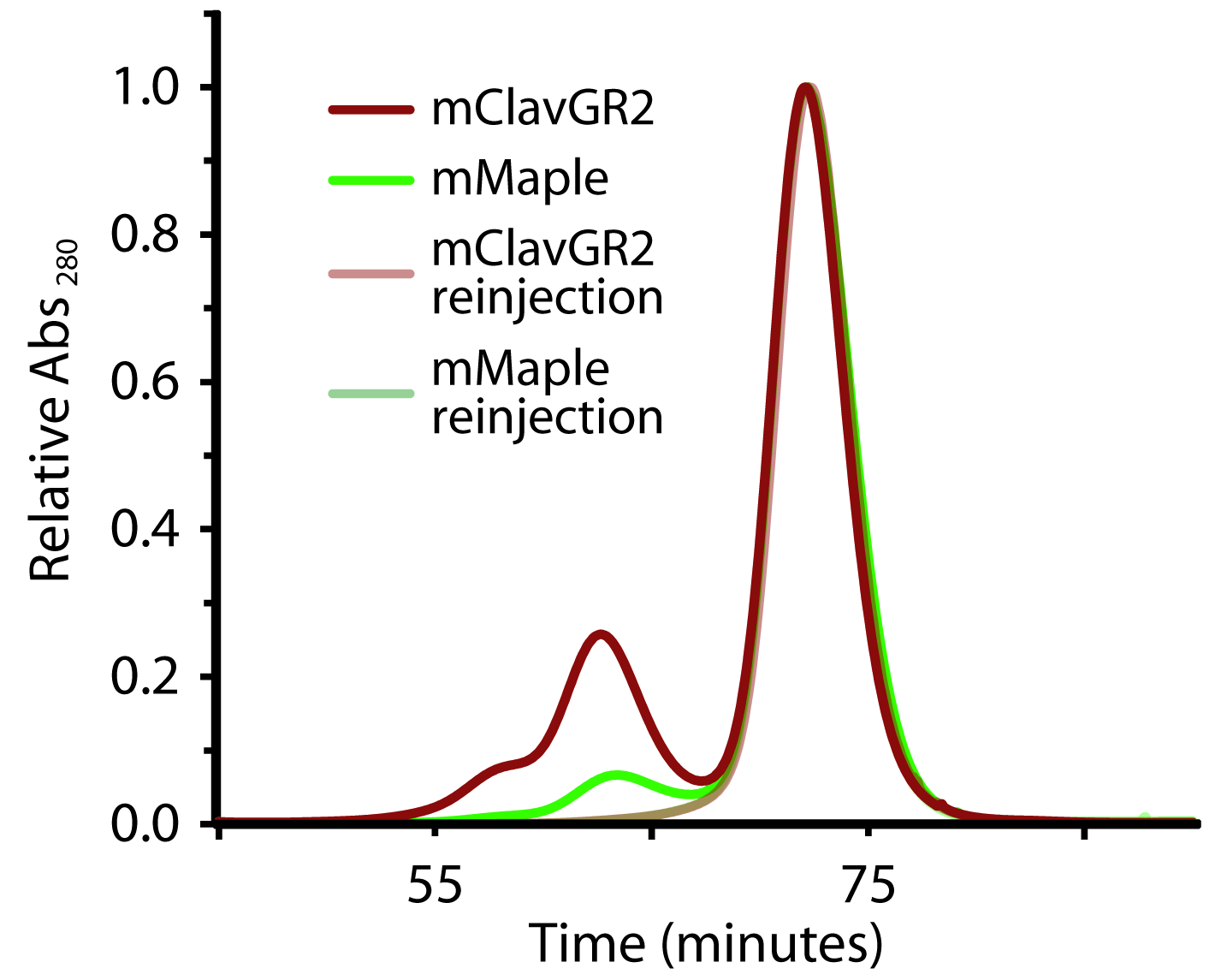

Supplement: Figure S1 — Gel filtration chromatography of mMaple and mClavGR2. Both mClavGR2 (injection concentration of 0.5 mM) and mMaple (injection concentration of 0.5 mM) purified from E. coli by Ni2+-NTA affinity chromatography show an additional peak at 63 min. This peak is diminished in size for mMaple relative to mClavGR2 (6.7% vs. 25.8% of monomer peak area). While this peak does elute at a time consistent with the dimer species, it was not observed following reinjection of the collected and concentrated monomeric peak of either mClavGR2 (injection concentration of 0.34 mM) or mMaple (injection concentration of 0.39 mM). The fact that the species eluting at 63 min was not observed in the reinjection suggests that it is not the typical non-covalent dimer species expected for weakly dimerizing fluorescent proteins. While the nature of this species remains unclear, it is apparent that the tendency of it to form is reduced in mMaple. (TIF) [file pone.0051314.s001.tif]

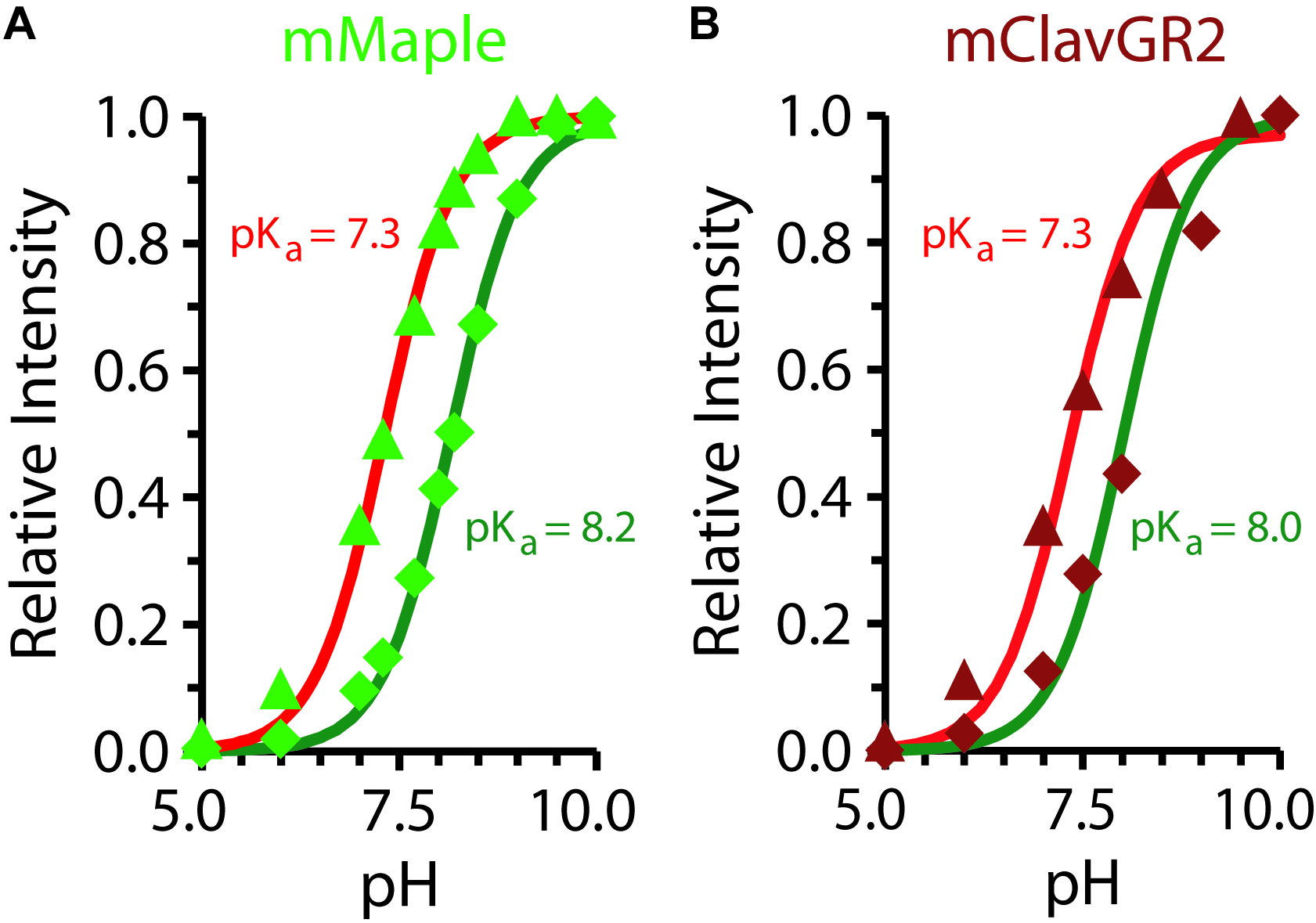

Supplement: Figure S2 — pH titrations of pcFP variants. For each variant the fluorescent intensity at pH values ranging from 5 to 10 was determined by diluting purified protein into concentrated buffer adjusted to the appropriate pH. For the green state (green line, diamond symbols), the λex = 440 nm and the λem = 530 nm. For the red state (red line, triangle symbols), the λex = 540 nm and the λem = 630 nm. (A) mMaple. (B) mClavGR2. (TIF) [file pone.0051314.s002.tif]

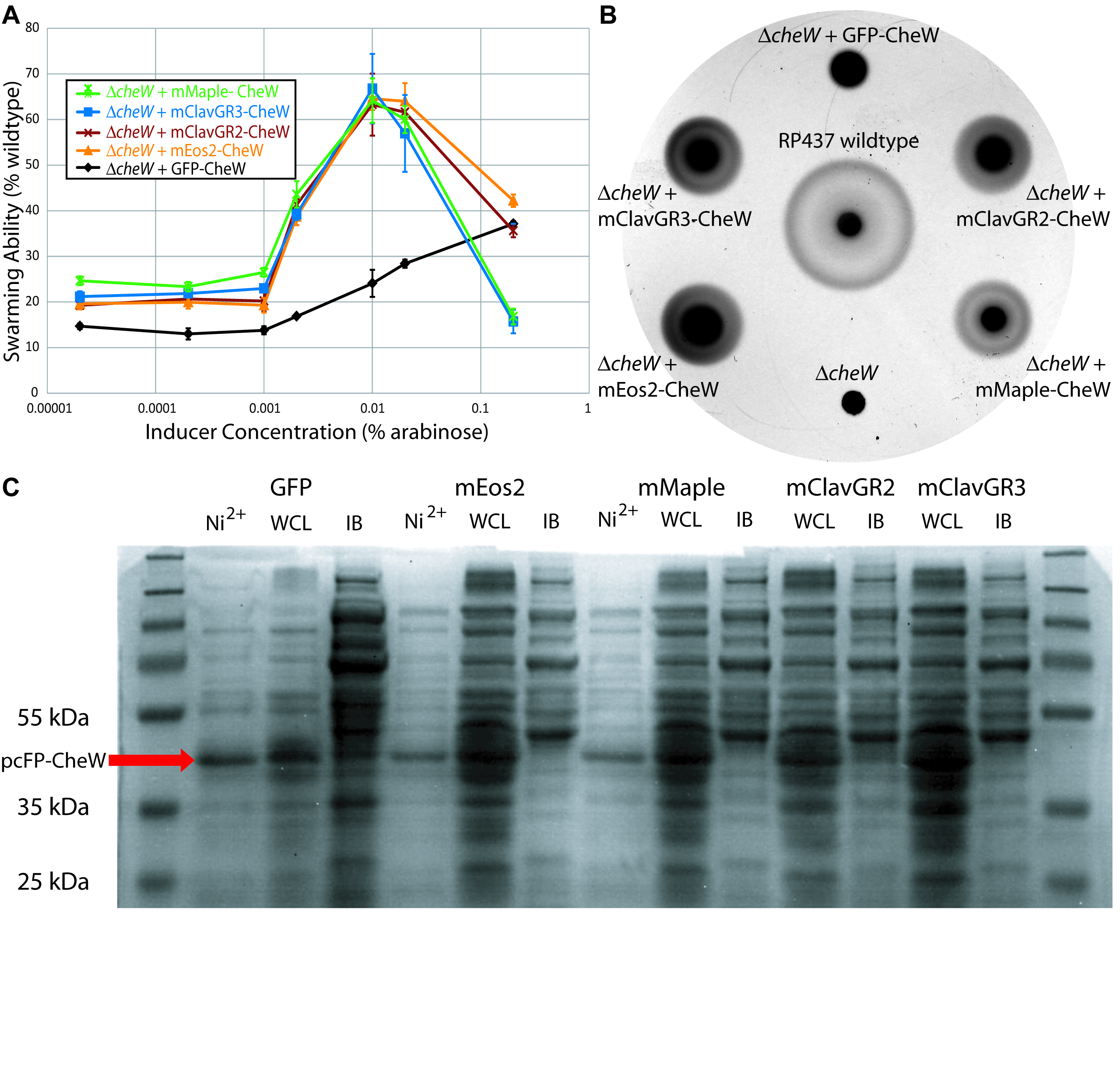

Supplement: Figure S3 — Swarm plate assays to assess the function of CheW fusions. Approximately 2 µl of ΔcheW E. coli transformed with a plasmid encoding a FP-CheW fusion were placed on T-broth soft agar swarm plates. The ability for the E. coli to undergo chemotaxis was assessed by measuring the diameter of the swarm ring 5 h after the bacteria were placed on the agar at 30°C. The ΔcheW strain has no apparent swarm ring and strain RP437 exhibits the wild-type swarm ring. Interestingly, fusions with all of the pcFPs used in this work are able to rescue the swarming phenotype more effectively than the analogous GFP fusion. (A) Swarming ability as a percentage of wild-type for bacteria expressing mMaple, mClavGR2, mEos2, or GFP fused to CheW. Excessively high concentrations of CheW can disrupt swarming ability, and thus the size of swarm rings will decrease at high inducer concentration [54]. Error bars are standard error, N = 3 measurements. (B) Image of a representative agar plate (0.01% L-arabinose concentration) showing swarm rings for each of the constructs mentioned above. (C) Coomassie stained SDS-PAGE gel of the soluble and insoluble fractions of E. coli expressing pcFP-CheWs (row denoted by red arrow) described in this work. First and last lanes are the protein ladder. Relative intensity of the bands is: 132, 120 and 27 for EGFP-CheW; 82, 52 and 0 for mEos2-CheW; 100, 164 and 0 for mMaple-CheW; 154 and 0 for mClavGR2; Samples were not denatured prior to loading. (TIF) [file pone.0051314.s003.tif]

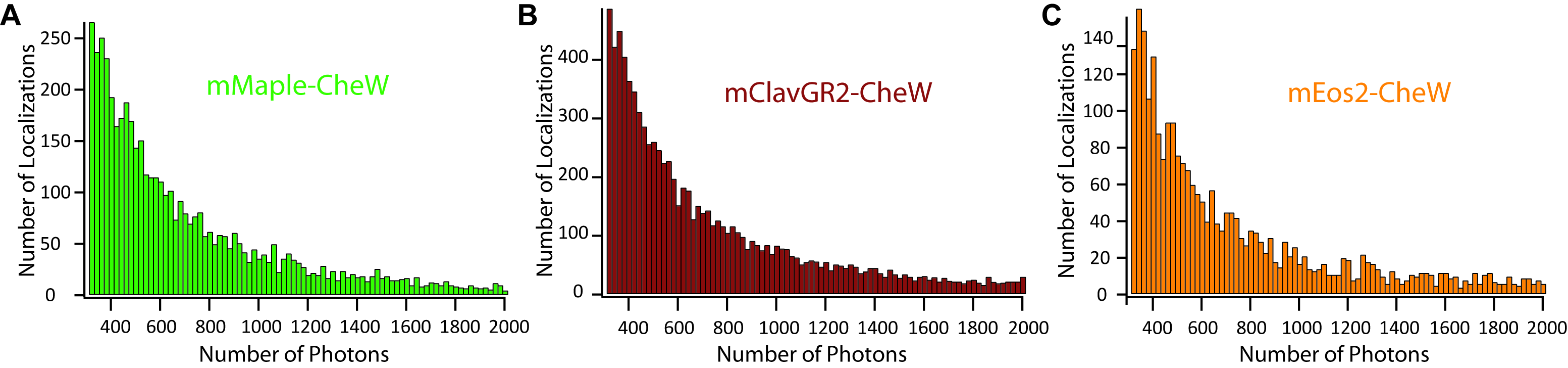

Supplement: Figure S4 — Number of photons emitted by pcFPs fused to CheW. Representative distributions of the number of photons emitted in the red fluorescent state by CheW fusions to (A) mMaple (B) mClavGR2 and (C) mEos2. Only localizations emitting more than 300 photons were included. (TIF) [file pone.0051314.s004.tif]

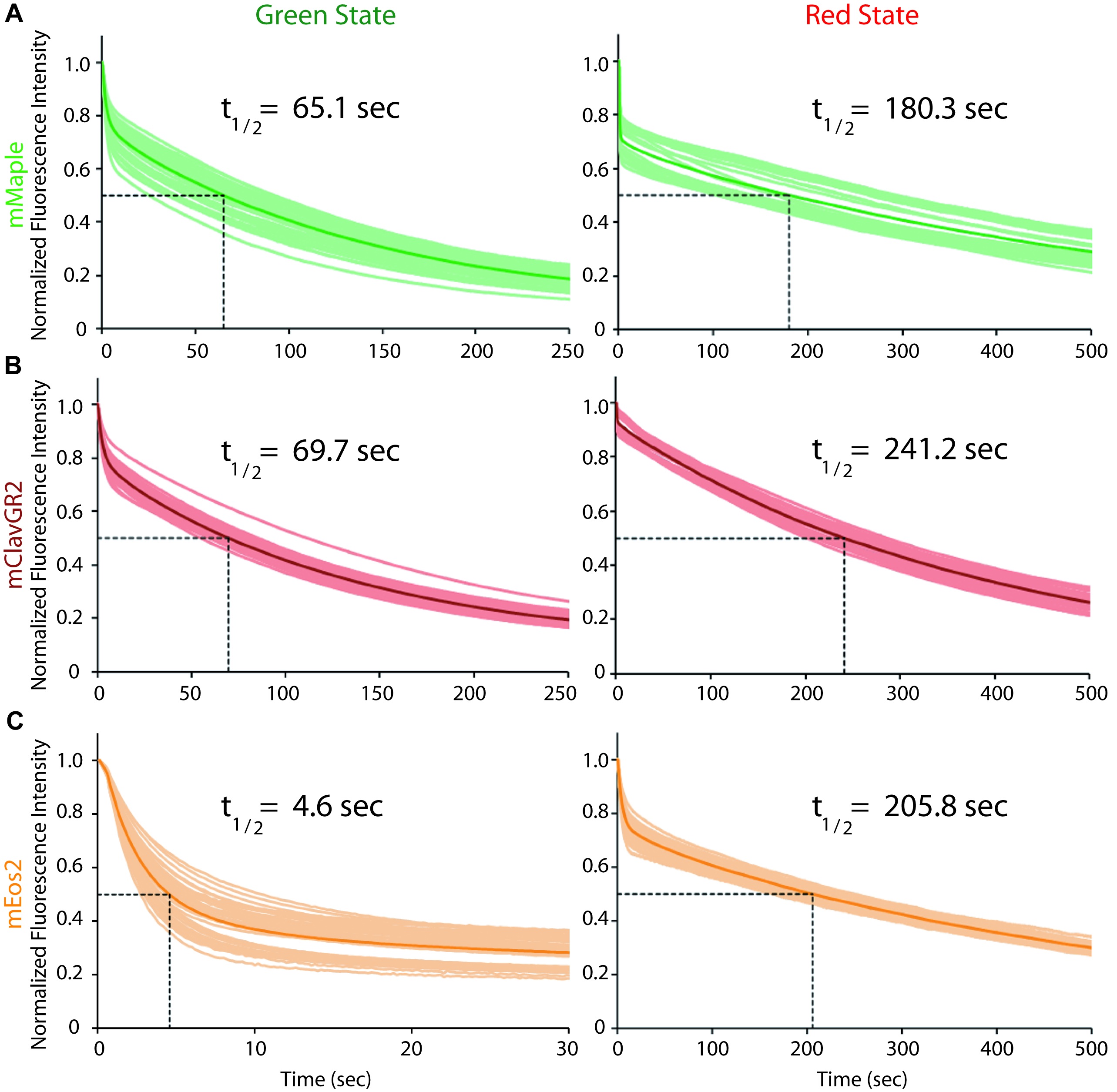

Supplement: Figure S5 — Widefield imaging photobleaching curves for pcFPs. Photobleaching curves of the green state (left panels) and the red state (right panels) of pcFP-H2B fusions expressed in HeLa S3 cells with widefield illumination. Each curve represents the photobleaching behavior of an individual cell and the darker colored curve is the average. Average time when the fluorescence intensity of the green states (left panels) decreased to half of the initial intensity are 65.1 sec for mMaple (A) (45 cells), 69.7 sec for mClavGR2 (B) (42 cells), and 4.6 sec for mEos2 (C) (48 cells). The average time for the red state is 180.3 sec for mMaple (A) (36 cells), 241.2 sec for mClavGR2 (B) (35 cells), and 205.8 sec for mEos2 (C) (49 cells). Values have been tabulated in Table S1. (TIF) [file pone.0051314.s005.tif]

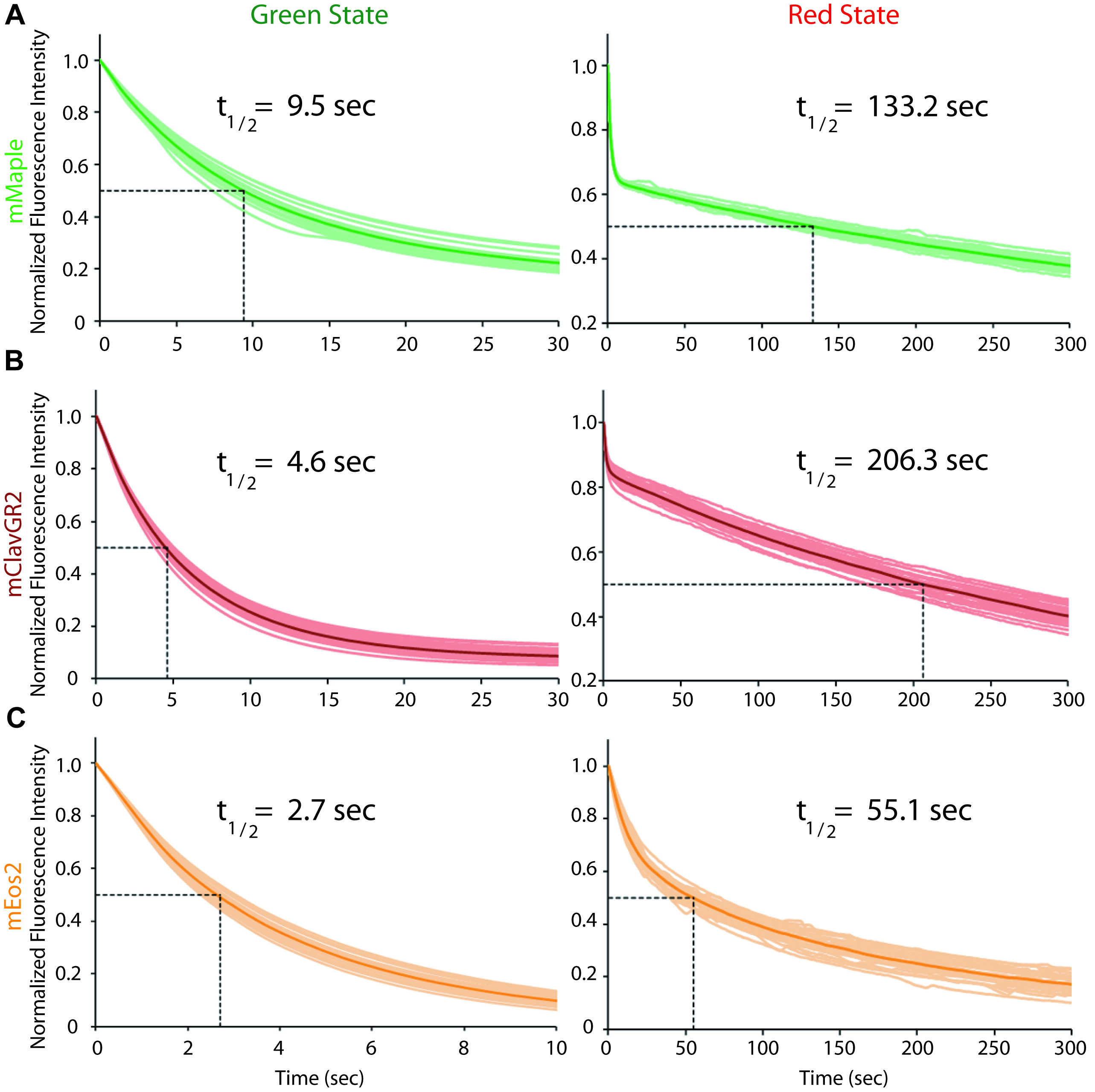

Supplement: Figure S6 — Confocal imaging photobleaching curves for pcFPs. Photobleaching curves of the green states (left panels) and the red states (right panels) of pcFP-H2B fusions expressed in HeLa S3 cells with confocal illumination. Each curve represents the photobleaching behavior of an individual cell and the dark colored curve is the average. Average time for the green state fluorescence intensity to decrease to half of the initial intensity are 9.4 sec for mMaple (A) (22 cells), 4.6 sec for mClavGR2 (B) (30 cells), and 2.7 sec for mEos2 (C) (28 cells). The average time for the red state fluorescence intensity to decrease by half is 133.2 sec for mMaple (A) (27 cells), 206.3 sec for mClavGR2 (B) (23 cells) and 55.1 sec for mEos2 (C) (25 cells). Values have been tabulated in Table S1. (TIF) [file pone.0051314.s006.tif]

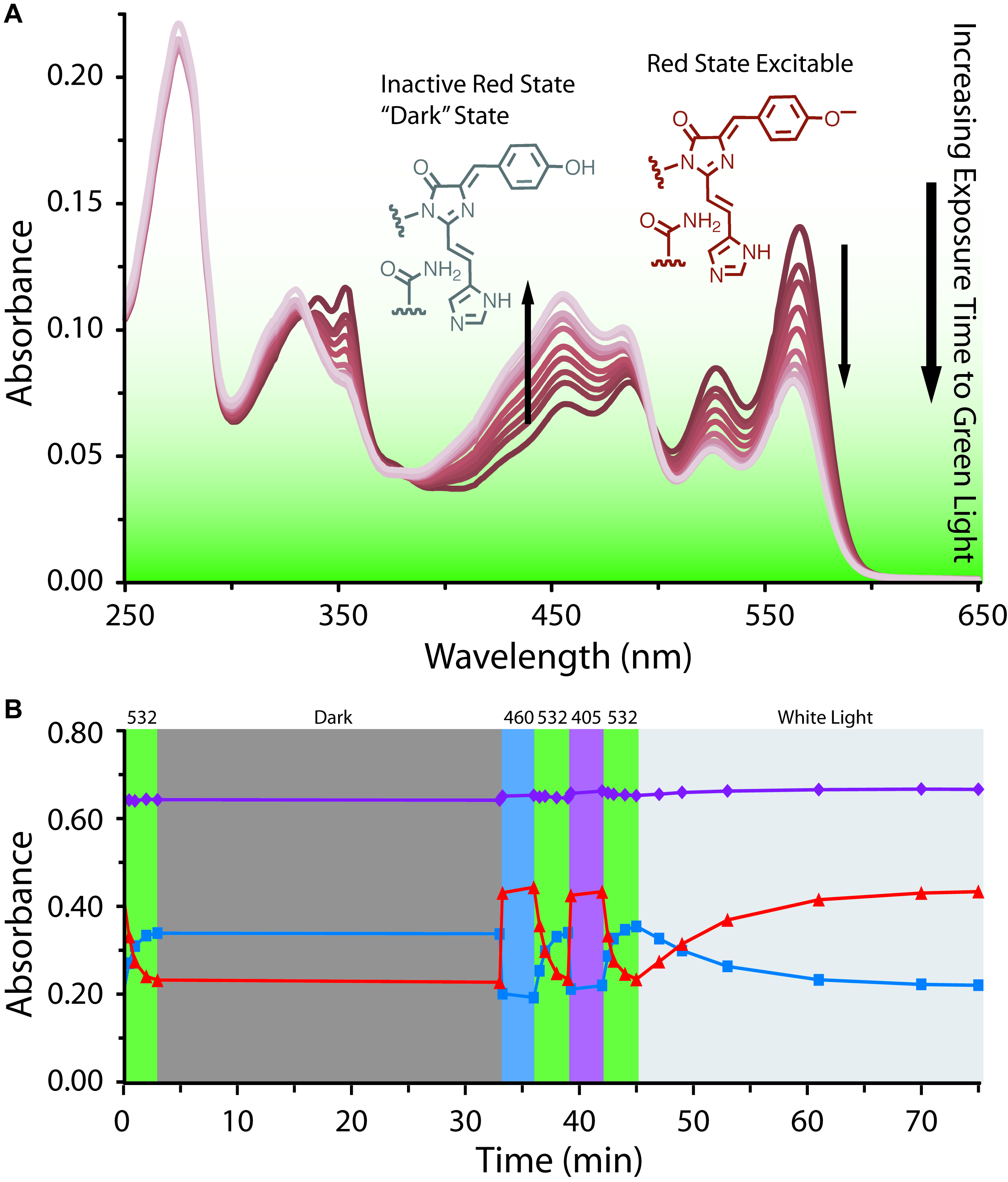

Supplement: Figure S7 — Reversible photoswitching of photoconverted (red) mMaple. (A) Photoconverted mMaple can be further photoconverted to a “dark” non-fluorescent state by illumination with green light. (B) The dark state of the photoconverted mMaple reversibly photoswitches back to the red fluorescent state as seen by changes in the absorbance spectra in response to different light sources. The absorbance at 280 nm (purple line, corresponding to total protein concentration), the absorbance at 457 nm (blue line, corresponding to the dark post-conversion red state) and the absorbance at 566 nm (red line, corresponding to the red fluorescent state of the protein) are plotted. As the protein is exposed to 532 nm light, the protein is switched from the red state to the dark state (green regions), which can be re-excited by 460 nm light (blue region), 405 nm light (violet region) and white light (light grey region). No absorbance changes were observed if the protein was kept in the dark (dark grey region). Similar results were obtained for mClavGR2. (TIF) [file pone.0051314.s007.tif]

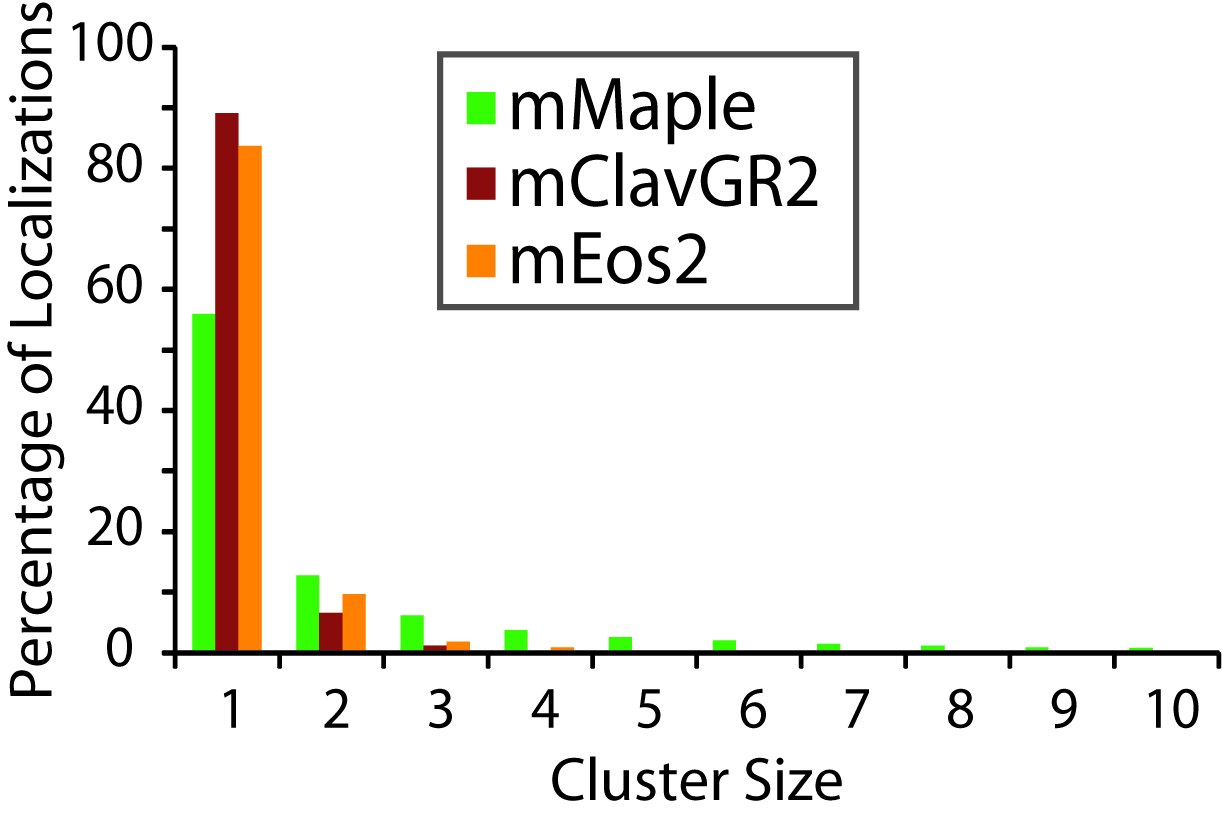

Supplement: Figure S8 — Cluster analysis of pcFP localizations. The percentage of localizations grouped into clusters (<30 nm interlocalization spacing) for cytoplasmically expressed pcFPs. Over 50% of mMaple and over 80% of mClavGR2 and mEos2 proteins do not have a second localization within 30 nm. (TIF) [file pone.0051314.s008.tif]

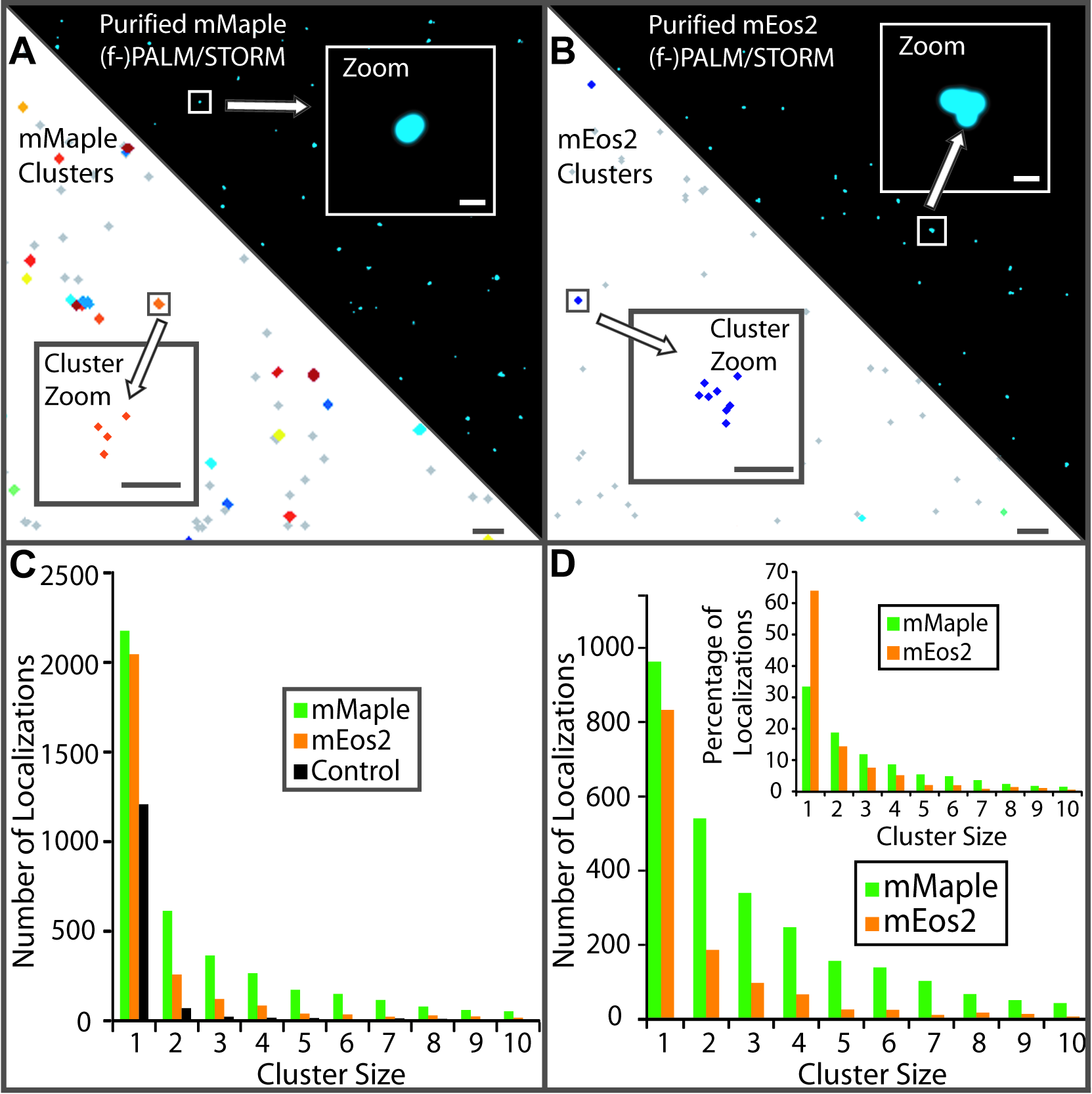

Supplement: Figure S9 — (f-)PALM/STORM analysis of purified pcFPs. (A–B) Composite images of purified (A) mMaple and (B) mEos2 proteins immobilized on a coverslip. Localizations are represented as normalized 2D Gaussian peaks (right half) and represented as single localizations and clustered markers (<30 nm interlocalization spacing) (left). Scale bars are 2 µm and 50 nm (zooms). (C) Cluster size distribution for purified mMaple and mEos2 as well as a no protein control. (D) False-positive corrected cluster size distributions for purified mMaple and mEos2 demonstrate that mMaple is approximately twice as likely to reactivate as mEos2. (inset) Percentage of false-positive corrected localizations found to be in clusters for purified pcFPs. 35% of mMaple and 65% of mEos2 do not have a second localization within 30 nm. (TIF) [file pone.0051314.s009.tif]

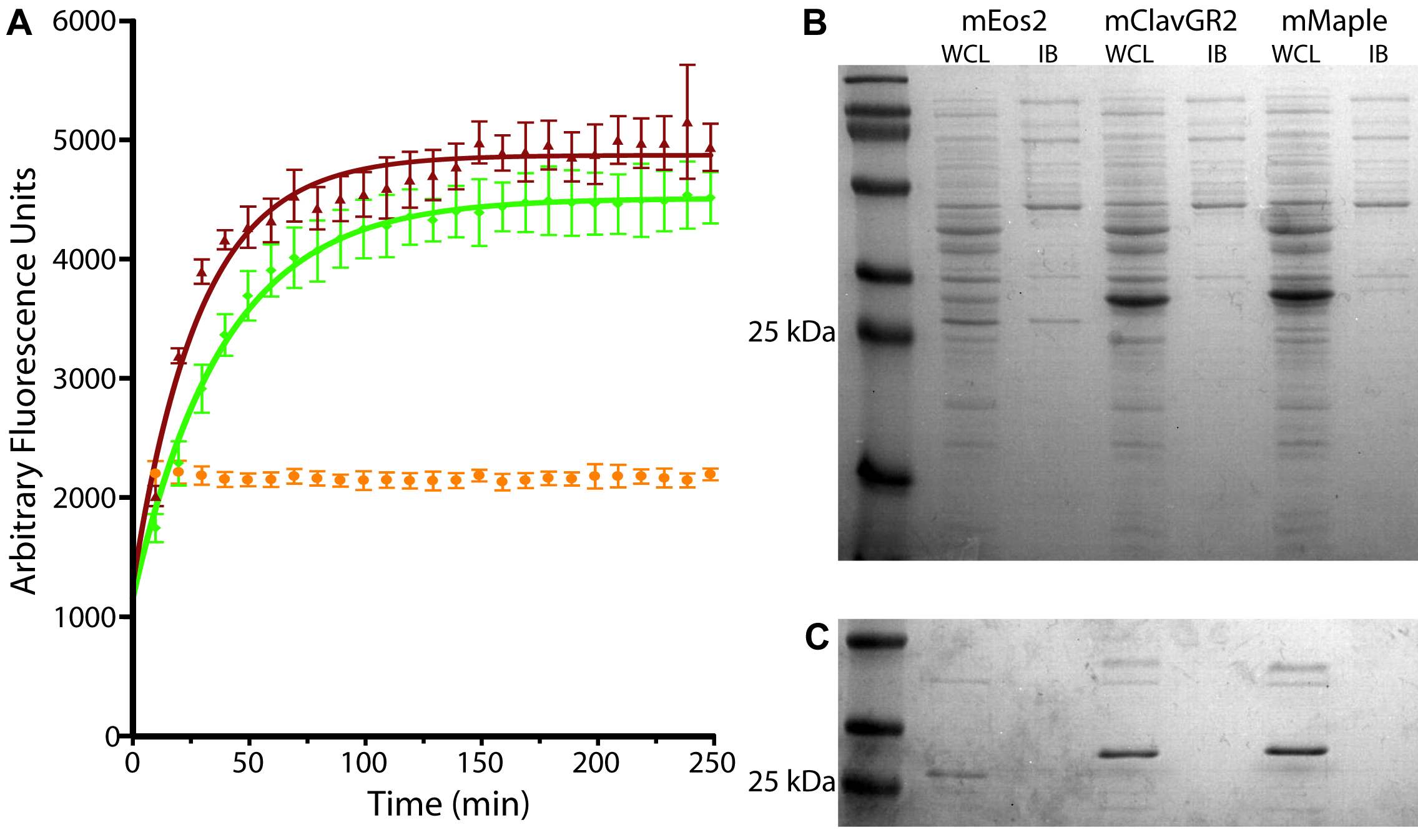

Supplement: Figure S10 — Expression and maturation of new pcFPs expressed in E . coli . (A) Maturation of mClavGR variants and mEos2 at 37°C. The maturation profiles of mMaple (green) and mClavGR2 (dark red) can be fit as monoexponential curves with time constants of 39 min and 29 min, respectively. Under the conditions of this experiment, mEos2 (orange) is approximately 50% as bright as mMaple and, in agreement with our previous results [31], appears to have fully matured prior to the initial measurement. Each curve represents the average of six independent measurements and error bars represent standard deviations. (B) SDS-PAGE of the soluble and insoluble fractions of E. coli expressing pcFPs described in this work. Lane 1 is the protein ladder. For each construct, one lane corresponds to the whole cell lysate (WCL) and the other lane corresponds to protein from inclusion bodies (IB). The relative intensity of FP bands in the WCL and IB fractions, respectively, are: 17 and 7 for mEos2; 100 and 0 for mClavGR2; 113 and 0 for mMaple. Overall, mMaple shows the highest expression and folding efficiency with 100% of the protein in the soluble fraction, while mEos2 has the lowest expression and folding efficiency with 29% of the total expressed protein located in the IB fraction. (C) The same samples as in (B), following purification by Ni2+/NTA affinity chromatography. (TIF) [file pone.0051314.s010.tif]

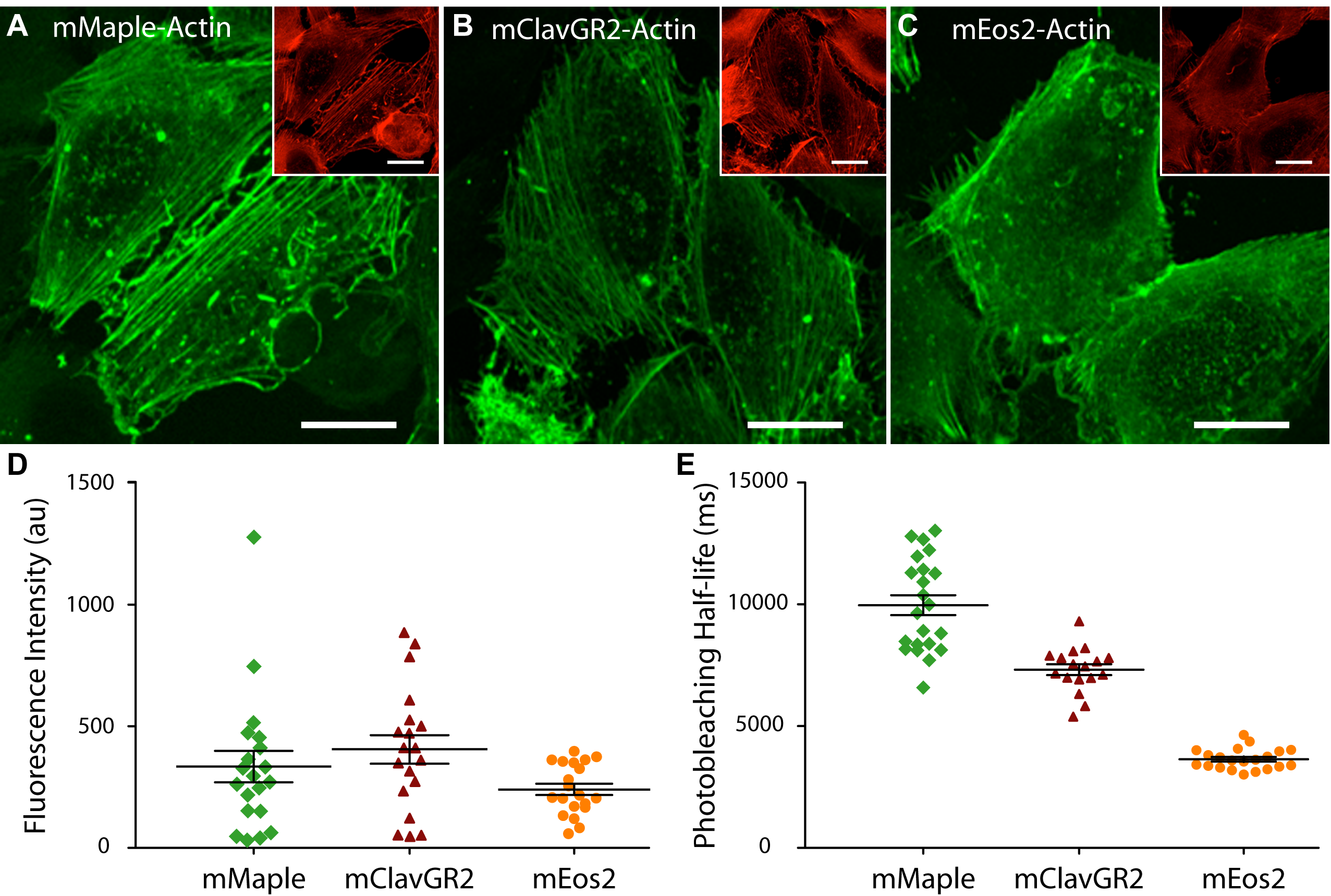

Supplement: Figure S11 — Photostability and green-state brightness characterization of pcFP-actin fusions in mammalian cells. Representative widefield fluorescence images of U2OS cells transfected with plasmids encoding either mMaple-actin (A), mClavGR2-actin (B), or mEos2-actin (C). The cells were stained with phallodin as a comparison (insets in A–C). (D) All pcFP-actin fusions have similar cellular intensities. However, mMaple is more photostable than the other two pcFP fusions (E). The data in (E) were found to be statistically significant using the Mann-Whitney t-test (p≤0.0001). (TIF) [file pone.0051314.s011.tif]
