# Supplementary material for: mMaple: A Photoconvertible Fluorescent Protein for Use in Multiple Imaging Modalities
Source: PLoS One. 2012 Dec 11;7(12):e51314. doi: 10.1371/journal.pone.0051314 (PMC3519878; doi:10.1371/journal.pone.0051314)
Supplement: Table S1 — Characterization of photobleaching rates for pcFP-H2B fusions in live cells. (DOC) [file pone.0051314.s012.doc]

|  |  | **Time to bleach to 50% of initial intensity (s)** | |
| --- | --- | --- | --- |
| **Protein name** | **State** | **Widefield imaging conditions^a^** | **Confocal imaging conditions^b^** |
| mMaple | green | 65.1 | 9.4 |
| mMaple | red | 180.3 | 133.2 |
| mClavGR2 | green | 69.7 | 4.6 |
| mClavGR2 | red | 241.2 | 206.3 |
| mEos2 | green | 4.6 | 2.7 |
| mEos2 | red | 205.8 | 55.1 |

^a^Widefield photobleaching of both states was performed at an output power of 11.4 mW/cm². ^b^Confocal photobleaching of both states was performed at an output power of 120 μW. All photobleaching curves are provided in Supplementary Fig. S4 and S5. We have previously reported [31] widefield (at 202.6 mW/cm² and 489.9 mW/cm² for green and red, respectively) and confocal (at 100 μW for both green and red) photobleaching times for mClavGR2 and mEos2 using units of “time to photobleach from 1000 to 500 photons/second/molecule”. We have since observed that the relative order of photobleaching half-times for different variants can change as a function of power. Accordingly, the numbers provided here are only relevant for the output power at which they were measured.
